# Angiopoietin-like protein 8 differentially regulates ANGPTL3 and ANGPTL4 during postprandial partitioning of fatty acids[S]

**DOI:** 10.1194/jlr.RA120000781

**Published:** 2020-06-02

**Authors:** Yan Q. Chen, Thomas G. Pottanat, Robert W. Siegel, Mariam Ehsani, Yue-Wei Qian, Eugene Y. Zhen, Ajit Regmi, William C. Roell, Haihong Guo, M. Jane Luo, Ruth E. Gimeno, Ferdinand van’t Hooft, Robert J. Konrad

**Affiliations:** * Lilly Research Laboratories, Eli Lilly and Company, Indianapolis, IN; † Division of Cardiovascular Medicine, Department of Medicine Solna, Karolinska Institutet Karolinska University Hospital Solna, Stockholm, Sweden

**Keywords:** lipoprotein lipase, adipose tissue, muscle, triglycerides, metabolic syndrome, angiopoietin-like protein 3, angiopoietin-like protein 4, lipid metabolism, obesity, postprandial condition

## Abstract

Angiopoietin-like protein (ANGPTL)8 has been implicated in metabolic syndrome and reported to regulate adipose FA uptake through unknown mechanisms. Here, we studied how complex formation of ANGPTL8 with ANGPTL3 or ANGPTL4 varies with feeding to regulate LPL. In human serum, ANGPTL3/8 and ANGPTL4/8 complexes both increased postprandially, correlated negatively with HDL, and correlated positively with all other metabolic syndrome markers. ANGPTL3/8 also correlated positively with LDL-C and blocked LPL-facilitated hepatocyte VLDL-C uptake. LPL-inhibitory activity of ANGPTL3/8 was >100-fold more potent than that of ANGPTL3, and LPL-inhibitory activity of ANGPTL4/8 was >100-fold less potent than that of ANGPTL4. Quantitative analyses of inhibitory activities and competition experiments among the complexes suggested a model in which localized ANGPTL4/8 blocks the LPL-inhibitory activity of both circulating ANGPTL3/8 and localized ANGPTL4, allowing lipid sequestration into fat rather than muscle during the fed state. Supporting this model, insulin increased ANGPTL3/8 secretion from hepatocytes and ANGPTL4/8 secretion from adipocytes. These results suggest that low ANGPTL8 levels during fasting enable ANGPTL4-mediated LPL inhibition in fat tissue to minimize adipose FA uptake. During feeding, increased ANGPTL8 increases ANGPTL3 inhibition of LPL in muscle via circulating ANGPTL3/8, while decreasing ANGPTL4 inhibition of LPL in adipose tissue through localized ANGPTL4/8, thereby increasing FA uptake into adipose tissue. Excessive caloric intake may shift this system toward the latter conditions, possibly predisposing to metabolic syndrome.

As humans evolved, the greatest survival threat was insufficient caloric intake (1–5). Our predecessors therefore relied on mechanisms that during times of starvation could direct FAs toward skeletal muscle to provide energy to hunt for food. Similarly, they relied on mechanisms that, during relatively rare periods of caloric availability, could reduce FA uptake into skeletal muscle and shift FA toward adipose tissue for storage as TGs to prepare for future periods of famine. These mechanisms allowed hominids to survive food scarcity. In a modern world of caloric abundance, however, the result is an unprecedented increase in metabolic syndrome and its related comorbidities (6–9).

Metabolic syndrome includes elevated TG, decreased HDL, obesity, hypertension, and insulin resistance/impaired glucose tolerance (9). This constellation of abnormalities predisposes not only to type 2 diabetes and increased cardiovascular disease but also to an increased risk of peripheral vascular disease, nonalcoholic fatty liver disease, and several types of cancer (10–17). It is currently estimated that two-thirds of the US population is overweight, and that one-third suffers from metabolic syndrome (18–23). At its most basic level, metabolic syndrome manifests as dysregulated lipid metabolism resulting in increased TG with excessive FA storage (as TG) in adipose tissue (24). Increased TGs are associated with decreased HDL, although the exact mechanisms have not been fully elucidated (25, 26). The excess adiposity causes insulin resistance (mainly in skeletal muscle through incompletely understood mechanisms) and hypertension (by increasing the arterial resistance) (27–37).

In the present study, we examine the role that angiopoietin-like protein (ANGPTL)8, a novel protein implicated in TG metabolism (38–48), plays in metabolic syndrome. ANGPTL8 is the most recently discovered member of the ANGPTL3/4/8 family of proteins involved in LPL regulation (39, 40, 45, 47, 49, 50). LPL is the enzyme responsible for conversion of TG (contained in lipoproteins) into FA that can be taken up into tissues such as skeletal muscle and fat (51–55). In general, increased LPL activity is thought to be beneficial as it would decrease circulating TG. ANGPTL3 and ANGPTL4 have been previously described as LPL inhibitors, and their inhibitory mechanisms have been at least partially characterized (42, 49, 56–66). In addition, ANGPTL3 has been shown to be an inhibitor of endothelial lipase, the enzyme that hydrolyzes phospholipids (PLs) in PL-rich HDL (56, 67).

ANGPTL3 knockout mice have been characterized as having decreased circulating TG (68, 69). In humans, ANGPTL3 knockout mutations are associated with decreased TG, decreased HDL (possibly due to increased endothelial lipase activity), and decreased LDL-C via unknown mechanisms (70–72). Humans with ANGPTL3 mutations have a reduced risk of cardiovascular events, presumably due to decreased TG and LDL-C (72, 73). ANGPTL4 knockout mice demonstrate decreased TG and an increased risk of intestinal lymphatic toxicity when placed on a high-fat diet (likely due to ANGPTL4 protecting against excessive saturated fatty acid uptake) (74–76). In humans, the ANGPTL4 E40K mutation has been associated with decreased TG, increased HDL, and a decreased risk of cardiovascular events (77–80). In a large human genetic study, review of medical records found no evidence of intestinal lymphadenopathy in 17 individuals homozygous for the ANGPTL4 E40K mutation, while no subjects were identified that were either homozygotes or compound heterozygotes for ANGPTL4 complete loss-of-function mutations (81).

ANGPTL8 was originally described as an atypical ANGPTL protein lacking the fibrinogen-like C-terminal domain present in other ANGPTL members (49). Subsequent reports showed that its overexpression in mice resulted in increased TG and that the effect was dependent upon ANGPTL3, indicating that the two proteins may work together in some way (39, 40). ANGPTL8 knockout mice were described as having decreased circulating TG (especially after refeeding) as well as reduced fat mass (41, 43). In humans, an ANGPTL8 knockout mutation has been associated with decreased TG, decreased LDL-C, and increased HDL. However, because the mutation is very rare, the study was not sufficiently powered to assess cardiovascular protection (82). Recently, Zhang (42) described an elegant ANGPTL3/4/8 model in which the three proteins are postulated to work together to move FA either toward the adipose tissue or skeletal muscle under feeding or fasting conditions, respectively. Using a mammalian expression system, Chi et al. (39) demonstrated that ANGPTL8 complexed with ANGPTL3, greatly enhanced the ability of ANGPTL3 to bind to and inhibit LPL, and required complex formation to be secreted efficiently. Very recently, Kovrov et al. (83) built upon these concepts by further exploring possible ideas for how ANGPTL8 might work together with both ANGPTL3 and ANGPTL4 to partition FA between adipose tissue and skeletal muscle.

In our current study, we examine the mechanisms by which ANGPTL8 acts as a key regulator of both ANGPTL3 and ANGPTL4 to direct FA toward adipose tissue after feeding. We show that in humans, ANGPTL8 increases with feeding and is present in ANGPTL3/8 and ANGPTL4/8 complexes, which can be measured in serum. Levels of these complexes correlate inversely with HDL and directly with all other markers of metabolic syndrome. In addition, we demonstrate that these complexes have dramatically opposite effects on LPL activity, with ANGPTL3/8 being over 100 times more potent than ANGPTL3 alone, while ANGPTL4/8 was more than 100-fold less potent than ANGPTL4 alone. We also show that ANGPTL4/8 can prevent ANGPTL3/8 from inhibiting LPL, thereby providing a mechanism to allow for LPL in the adipose tissue to be protected from increased postprandial circulating ANGPTL3/8 levels. Together, our data demonstrate how increased ANGPTL8 levels that occur following feeding can decrease LPL activity in the skeletal muscle while increasing LPL activity in the fat, thus directing postprandial uptake of FA into adipose tissue.

## MATERIALS AND METHODS

### Normal human serum samples and SCARF control samples

Sera were obtained (with consent) from healthy volunteers from the Eli Lilly Research Blood Donor Program. To study fasting and postprandial conditions, sera were obtained from 10 healthy volunteers after overnight fasting and 1 and 2 h following a mixed-meal breakfast consisting of approximately 400 carbohydrate calories, 400 fat calories, and 100 protein calories. All samples were stored at −80°C. SCARF is a case-control study from northern Stockholm comprising consecutive, unselected MI survivors below age 60 and controls matched for age, sex, and area of residence (84–86). The study was approved by the Ethics Committee of the Karolinska University Hospital and conducted in agreement with the Declaration of Helsinki. All subjects gave informed consent to participate. Control subjects were interviewed regarding lifestyle characteristics, medical history, and medication, and a physical examination was performed. Samples were collected under fasting conditions and stored at −80°C. The Clinical Chemistry Laboratory of the Karolinska University performed standard serum analyses (84).

### Recombinant ANGPTL protein and complex generation

Human sequences were as follows: ANGPTL8, NP_061157.3; ANGPTL3, NP_055310.1; and ANGPTL4, NP_647475.1. Mature ANGPTL8 (residues 22-198) was produced in *Escherichia coli* as inclusion bodies and refolded in vitro. C-terminal HIS-tagged ANGPTL4 and ANGPTL3 were produced stably in CHO cells and transiently in HEK293 cells, respectively. Both were purified through nickel-nitrilotriacetic acid (Ni-NTA) affinity, followed by size exclusion chromatography (SEC). ANGPTL3/8 complex was produced in HEK293 cells through transient cotransfection. Nucleotide sequences encoding mouse IgG kappa signal peptide-HIS tag-mature human serum albumin (HSA)-PreScission cleavage site-mature ANGPTL8 were inserted into a mammalian expression vector containing a cytomegalovirus promoter, as were the nucleotide sequences encoding C-terminal Flag-tagged ANGPTL3. Protein expression was performed through transient cotransfection of both expression constructs in HEK293 cells cultured in serum-free media. Culture media were harvested 5 days post transfection and stored at 4°C for subsequent protein purification at 4°C. Four liters of culture media were supplemented with 1 M Tris-HCl (pH 8.0) and 5 M NaCl to final concentrations of 25 mM and 150 mM, respectively. The media were incubated with 150 ml of Ni-NTA resin (Qiagen) overnight. The resin was then packed into a column and washed with buffer A [50 mM Tris-HCl (pH 8.0), 0.3 M NaCl]. Elution was performed with a 0–300 mM imidazole gradient in buffer A. Fractions containing HIS-HSA-ANGPTL3/8 complex were pooled, concentrated, loaded onto a HiLoad Superdex 200 column (GE Healthcare), and eluted with buffer A. Fractions containing HIS-HSA-ANGPTL3/8 were again pooled, concentrated, and digested with PreScission protease to remove HSA from the HIS-HSA-ANGPTL8 fusion protein. The PreScission-digested protein sample was loaded onto another HiLoad Superdex 200 column and eluted with storage buffer [20 mM HEPES (pH 8.0), 150 mM NaCl]. Fractions containing ANGPTL3/8 complex were pooled and concentrated. Protein concentrations were determined using a BCA protein assay.

During the ANGPTL3/8 purification process, it was important for the ANGPTL3/8 complex not to contain free proteins. To ensure purity, the initial Ni-NTA affinity purification first removed all free ANGPTL3. After SEC, purified HIS-HSA-ANGPLT3/8 complex and free HIS-HSA-ANGPLT8 were obtained. PreScission digestion (which cleaved between HSA and ANGPTL8) resulted in ANGPTL3/8 complex, HIS-HSA, and free ANGPTL8. Free ANGPTL8 was precipitated out, leaving only ANGPTL3/8 complex and HIS-HSA. ANGPTL3/8 complex and HIS-HSA were separated with a second SEC step, resulting in highly purified ANGPTL3/8 complex without any HIS-HSA contamination (as shown in Fig. 2A). This strategy ensured that very pure ANGPTL3/8 complex was produced. The same approach was used for expression and purification of the ANGPTL4/8 complex. All proteins and complexes were maintained at a <0.01 EU/μg of endotoxin. One microgram of each recombinant ANGPTL protein or complex was characterized using gradient gel electrophoresis with Bio-Rad 4–20% Mini-Protean Tris-glycine gels, followed by Coomassie Blue staining to verify the purity of the respective proteins and complexes, which were all stored at −80°C. For purposes of molar conversions, a molecular mass of 179 kDa was used for ANGPTL3/8 (3:1 ratio), while a molecular mass of 64 kDa was used for ANGPTL4/8 (1:1 ratio).

### ANGPTL antibodies

Anti-human ANGPTL8 antibodies (residues 22-198) were generated using hybridoma techniques by Precision Antibody Sciences. Anti-human ANGPTL4 antibodies were generated by immunization with recombinant ANGPTL4 (residues 26-161) or purchased commercially (R&D Systems, AF3485). Anti-human ANGPTL3 antibodies were generated after immunization with mammalian produced recombinant ANGPTL3 (residues 17-220) or purchased commercially (R&D Systems, AF3829). Clones of interest were screened for nonoverlapping epitopes, and antigen-specific variable heavy and variable light gene sequences were determined from extracted RNA using a mouse Ig primer set (EMD Millipore). Variable domains were transferred into separate murine constant region expression vectors for antibody production, transfected into CHO cells, and purified using protein A chromatography. Antibodies were biotinylated using a Pierce kit and ruthenium-labeled using a Meso Scale Discovery (MSD) kit, with MALDI-TOF performed to verify appropriate labeling. Antibodies were diluted in 50% glycerol and stored at −20°C.

### Immunoprecipitation/Western blotting

Anti-ANGPTL and control antibodies were covalently coupled to tosyl-activated beads (Thermo), with heavy and light chains further cross-linked using dimethyl pimelimidate. Fifty microliters of beads containing 20 μg of antibody were added to 4 ml of pooled donor serum diluted 1:2 with PBS and incubated at 4°C overnight. Beads were washed with PBS and boiled in sample buffer. Proteins were separated on a Novex 12% Bis-Tris gel and transferred to PVDF using an iBlot system (Thermo). Membranes were probed with biotinylated anti-ANGPTL antibodies. Visualization was performed with Alexa Fluor 680-conjugated streptavidin. Images were recorded using an Odyssey CLx image system (LI-COR Biosciences).

### Immunoprecipitation-mass spectrometry

Proteins were immunoprecipitated from normal human serum using anti-ANGPTL8, -ANGPTL4, or -ANGPLT3 antibodies. The anti-ANGPTL4 antibody utilized was an N-terminal antibody. Irrelevant IgG was used as a negative control. Biotinylated antibodies (10 μg) were added to 1 ml of human serum diluted with 1 ml of PBS, and samples were incubated at 4°C overnight. The next day, 30 μl of streptavidin magnetic beads (Thermo) were added, and tubes were incubated at 4°C for 2 h. Beads were washed using PBS, and bound proteins were reduced with DTT and alkylated with iodoacetamide. Proteins were digested using 1 μg of Trypsin/Lys-C (Promega) at 37°C for 4 h. Digests were acidified using 5 μl of 10% trifluoroacetic acid. Stable-isotope-labeled (SIL) peptides (0.2 pmole) were spiked into each sample before analysis with a TSQ Quantiva (Thermo) using LC-multiple reaction monitoring (MRM). Peptides were separated using a Hypersil Gold C18 HPLC column (50 × 2.1 mm) with a Dionex Ultimate 3000 system at a flow rate of 250 μl/min. Solvent A consisted of 0.1% formic acid in water, and solvent B consisted of 0.1% formic acid in acetonitrile. For protein quantification, SIL peptides for each ANGPTL protein were synthesized with selected lysine or arginine residues labeled with ^13^C and ^15^N, and peptide content of SIL peptides was determined through amino acid analysis. Peak area ratios between the endogenous and corresponding SIL peptides were used to estimate protein concentrations after averaging results from two peptides for each protein analyzed. The specific SIL peptides used for quantitation are listed in supplemental Figs. S1–S7.

### Mass spectrometry assessment of ANGPTL complexes

ANGPTL3/8 and ANGPTL4/8 were digested at 37°C for 4 h using a mixture of trypsin/Lys-C, after reduction with DTT and alkylation with iodoacetamide. Two peptides from each protein were used for quantitation as described in supplemental Figs. S1–S7 and supplemental Tables S1–S4. For LC-MRM quantification, an identical molar amount of each SIL peptide was added to the protein digest, and samples were analyzed using a TSQ Quantiva triple quadrupole mass spectrometer. Xcalibur software (version 4.2.47, Thermo) was used to determine peak area ratios between the digested peptide and its corresponding SIL peptide. Detailed peak integration parameters used for the analysis are included in supplemental Figs. S1–S7. Individual protein ratios in ANGPTL3/8 and ANGPTL4/8 were calculated using the average ratio from two SIL peptides for each protein in each complex. To assess the protein ratios of endogenous complexes, ANGPTL3/8 and ANGPTL4/8 were immunoprecipitated from human serum. Molar ratios for ANGPTL3:ANGPTL8 and ANGPTL4:ANGPTL8 were then calculated using spiked-in SIL peptide standards utilizing the methods described above for the recombinant complexes.

### Mass spectrometry experimental design and rationale

Peptides with robust ionization after tryptic digest analysis of ANGPTL proteins were selected for MRM experiments. Protein BLAST (https://blast.ncbi.nlm.nih.gov/Blast.cgi) searches against the NCBI human database (the program’s default) confirmed that these peptides were unique to the corresponding protein, with no known posttranslational modifications. At least two transitions were selected for each peptide for MRM monitoring. The amino acid sequences of these peptides and specific transitions are listed in supplemental Tables S1–S4. The transitions selected, the collision energy, and RF lens values were optimized by infusing the synthesized SIL peptides. No significant interference was detected at the corresponding retention time for each peptide in the negative controls.

### ANGPTL immunoassays

We used dedicated immunoassays to measure each protein or complex of interest. For the ANGPTL8 assay, monoclonal antibodies directed against independent ANGPTL8 epitopes were used for capture and detection. Based on our mass spectrometry and immunoassay-based quantitation of human serum ANGPTL8, ANGPTL3/8 complex, and ANGPTL4/8 complex, ANGPTL8 measured by this assay was mainly present in either ANGPTL3/8 or ANGPTL4/8 complexes; however, we could not rule out the possibility that a small amount of free ANGPTL8 might also circulate. For ANGPTL3, a monoclonal antibody was used for capture and a polyclonal anti-ANGPTL3 antibody (R&D Systems, AF3829) was utilized for detection. The total level of ANGPTL3 was found to be much greater than that of the ANGPTL3/8, suggesting that the vast majority of ANGPTL3 detected by this assay was free ANGPTL3. An assay employing two different monoclonal antibodies recognizing independent N-terminal epitopes of ANGPTL4 was used to measure active ANGPTL4 (defined as full-length ANGPTL4 or the N-terminal fragment of ANGPTL4). For measurement of the C-terminal domain-containing (CTDC) ANGPTL4 (defined as full-length ANGPTL4 or the inactive C-terminal fragment of ANGPTL4), an R&D Systems kit (DY3485) was utilized. For the ANGPTL3/8 assay, the capture antibody recognized ANGPTL8, and the detection antibody recognized ANGPTL3. For the ANGPTL4/8 assay, the capture antibody recognized ANGPTL4, and the detection antibody recognized ANGPTL8. The ANGPTL4/8 complex assay could not distinguish between the N-terminal ANGPTL4 fragment and the full-length ANGPTL4 present in the ANGPTL4/8 complex.

For each assay, MSD streptavidin plates were washed three times with TBST [TBS containing 10 mmol/l Tris (pH 7.40), 150 mmol/l NaCl, and 1 ml/l Tween 20]. Plates were blocked with TBS plus 1% BSA for 1 h at RT. After aspiration and washing, wells were incubated with biotinylated capture antibody for 1 h. Following aspiration and washing, 50 μl of recombinant protein or complex (serially diluted to form a standard curve) were added to the wells in assay buffer [50 mmol/l HEPES (pH 7.40), 150 mmol/l NaCl, 10 ml/l Triton X-100, 5 mmol/l EDTA, and 5 mmol/L EGTA]. Serum samples were diluted in assay buffer and added to their respective wells for a 2 h incubation at RT. After aspiration, wells were washed three times, and 50 μl of ruthenium-labeled detection antibody were added for a 1 h incubation at RT. Following aspiration, wells were washed three times, and 150 μl of MSD read buffer were added. Electrochemiluminescence from electrical excitation of ruthenium in the wells was detected using an MSD plate reader. Specificity of each novel assay was tested using the other proteins/complexes, and each had cross-reactivity of less than 1%.

### VLDL-C uptake assay

We adopted the method of Neher and colleagues (87). In this assay, VLDL particles are labeled with a fluorescent PL probe, which is inserted into the outer PL layer of the VLDL particle, and the assay measures the adherence of the fluorescence probe to Huh7 cells. For labeling of VLDL (Lee Solutions) with 1,2-dioleoyl-*sn*-glycero-3-phosphoethanolamine-*N*-carboxyfluorescein (18:1 PECF; Avanti), 100 μl of PECF (1 mg/ml in chloroform) was dried under argon. Next, 400 μl of VLDL (10 mg/ml TG, in PBS) were added to the vial, vortexed, and sonicated for 6 min to yield final concentrations of 10 mg/ml TG VLDL and 0.25 mg/ml PECF. Huh7 cells were plated at a density of 40,000 cells/well in a poly-D-lysine 96-well plate in medium consisting of DMEM/F-12 3:1 (Gibco), 10% FBS (Gibco), 1% penicillin-streptomycin (Gibco), and 20 mM HEPES (Gibco). Cells were grown overnight before medium was replaced with 150 μl of PBS for 2.5 h. LPL (Sigma) was mixed with ANGPTL3/8 or ANGPTL4/8 and incubated at RT for 1 h with gentle shaking. Mixtures were combined with equal volumes of VLDL-PECF (final concentrations of 11 U/ml LPL, 50 nM ANGPTL3/8 or ANGPTL4/8, 100 μg/ml VLDL-PECF), and 50 μl of the mixtures replaced the PBS. Cells were incubated at 37°C for 30 min. Media were then replaced with 150 μl/well of fixative. Cells were fixed for 20 min, washed twice with 200 μl of PBS, and covered with 50 μl/well of PBS. Fluorescence at 495/525 nm was measured using a SpectraMax3 plate reader, with VLDL uptake calculated as relative fluorescent units at 525 nm.

### Binding assessments

ANGPTL interactions with LPL were assessed with bio-layer interferometry using Octet RED96e® (Molecular Devices). Avidin-tagged LPL was immobilized on streptavidin biosensors. Immobilized LPL (Sigma) was incubated with 50 nM of ANGPTL3, ANGPTL3/8, ANGPTL4, or ANGPTL4/8 and transferred into buffer-only wells to monitor dissociation.

### LPL stable expression cell line and activity assays

The nucleotide sequence for human LPL (NP_000228.1) was inserted into pLenti6.3 vector (Invitrogen) to generate lentivirus, which was used to create a stable expression cell line confirmed by qPCR and enzymatic activities. The cell line was maintained in DMEM/F12 (3:1) (Invitrogen), 10% FBS (Hyclone), and 5 μg/ml blasticidin (Invitrogen). The wild-type human LPL-stable expression cells were seeded at a density of 50,000 cells/well in tissue culture-treated 96-well plates (Costar) in growth medium (3:1 DMEM/F12, 10% FBS, and 5 μg/ml blasticidin). After overnight incubation, medium was replaced with 80 μl of medium containing serially diluted ANGPTL proteins. Cells were incubated for 1 h before 20 μl of 5× working solution, freshly prepared with 0.05% Zwittergent detergent 3-(*N*,*N*-dimethyl-octadecylammonio)-propanesulfonate (Sigma) and containing EnzChek lipase substrate BODIPY-dabcyl-labeled TG analog (Invitrogen), were added to achieve a final concentration of 1 μM (88). Fluorescence was monitored at 1 and 30 min with a Synergy Neo2 plate reader with an excitation wavelength of 485 nm and emission wavelength of 516 nm to correct for background. To study the ability of ANGPTL4/8 to protect LPL from ANGPTL3/8 and ANGPTL4 inhibition, ANGPTL4/8 was first serially diluted in growth medium, and 60 μl of the medium containing ANGPTL4/8 were added to the cells for a 1 h incubation with gentle shaking. Afterward, 20 μl of 5 nM ANGPTL3/8 or ANGPTL4 working solution (5×) prepared in growth medium were added for a further 1 h incubation. Finally, 20 μl of EnzChek lipase substrate were added to start the reaction. Fluorescence was monitored at 1 and 30 min with a Synergy Neo2 plate reader with an excitation wavelength of 485 nm and emission wavelength of 516 nm to correct for background.

### LPL activity assay with VLDL as substrate

The assay was similar to those described above, except that Enzchek lipase substrate was replaced with 20 μl/well of (Lee Solutions) VLDL (2 mM TG, final 0.4 mM TG in the well), and NEFAs released by LPL were measured using an NEFA-HR kit (Wako). Human LPL-stable expression cells were seeded at 50,000 cells/well in a poly-D-lysine-coated 96-well plate. Cells were grown overnight, and medium was replaced with 80 μl of medium containing serially diluted ANGPTL4 or ANGPTL4/8 complex. Cells were incubated for 1 h with gentle shaking. Twenty microliters of VLDL (2 mM TG) were added to each well, and the plate was incubated for 30 min, after which 5 μl of medium were used for NEFA measurement.

### Samples from patients treated with hepato-preferential insulin

Basal insulin peglispro (BIL) is a pegylated version of insulin lispro with a large dynamic radius that restricts it from the periphery but allows it to pass through hepatic sinusoids, thus making it hepato-preferential (89). Clinical trial samples were obtained from a previously described study in which insulin-naïve patients were administered BIL for 52 weeks (90). These samples were from 279 patients at baseline and during 52 weeks of BIL treatment (all drawn under morning fasting conditions). Ideally, we would have analyzed samples from early time points; however, the only postbaseline sera available were those collected after 12, 26, and 52 weeks of treatment. Samples were stored at −80°C prior to analyses.

### Secretion of ANGPTL complexes from hepatocytes

Human primary hepatocytes were obtained from BioIVT in the HepatoPac platform. Cells were incubated for 2 days in BioIVT maintenance media. Following aspiration, cells were washed in serum-free BioIVT application media. Afterward, cells were preincubated for 1 day in application media in the absence of insulin, and then incubated overnight with application media in the absence or presence of 1 nM insulin. Media were collected and stored at −80°C prior to analyses.

### Effect of dextran sulfate on ANGPTL protein release

C-terminal Flag-tagged ANGPTL4 and C-terminal HIS-tagged ANGPTL8 mammalian expression constructs were transfected into HEK293 cells. At 24 h posttransfection, 0 or 0.1 mg/ml of dextran sulfate was added to the media. The media were harvested 5 days posttransfection, and equal volumes from each treatment condition were used for immunoblotting with either anti-Flag or anti-HIS antibody.

### Adipocyte experiments

Human adipose-derived stem cells were obtained from Zen-Bio and seeded in 96-well plates (160,000 cells/cm^2^ in 100 μl of EGM2-MV media; Zen-Bio). After 24 h, media was replaced by PM1 (Zen-Bio) followed by replacement with DM2 (Zen-Bio) media. Fresh DM2 was added on the third day followed by replacement with AM1 (Zen-Bio) every 3 days until the cells were used. Differentiated adipocytes were utilized between days 12 and 14. For adipocyte mRNA analyses, differentiated cells were treated for 8 h in PM1 in the absence or presence of 100 nM of insulin (Sigma) in DMEM/F12 (3:1) (Gibco) containing 0.2% fatty acid-free BSA (Thermo). RNA was extracted using a miRNeasy mini kit (Qiagen). One microgram of total RNA was reverse transcribed using a high capacity cDNA kit (Applied Biosystems). The cDNA was diluted 1:10, and ANGPTL4 (Applied Biosystems; TaqMan mix Hs01101127_m1) and ANGPTL8 (Applied Biosystems; TaqMan mix Hs00218820_m1) transcript levels were quantitated. For insulin-stimulated release of ANGPTL4/8 complex, adipocytes were cultured in DMEM (Gibco) containing 0.2% fatty acid-free BSA (Thermo). Cells were treated overnight in media containing 20 units/ml heparin (Sigma) with 0–100 nM of insulin in the absence and presence of 10 nM of glucose-dependent insulinotropic peptide (GIP). Media were collected and stored at −80°C prior to analyses.

### Statistics

For SCARF samples, variables that presented skewed distribution were logarithmically transformed, and associations between ANGPTL complexes and selected phenotypes were assessed using Spearman rank correlation coefficients. A four-parameter logistic nonlinear regression model was used to fit curves for LPL activity assays, while MSD software was used for immunoassay calibration curves. Significance for the feeding effect on ANGPTL complexes was assessed using a paired *t*-test. Significance for the effect of complexes on VLDL-C uptake and the effect of insulin on adipocyte ANGPTL4/8 secretion was assessed using an unpaired *t*-test. A Dunnett’s test was used for insulin stimulation of ANGPTL mRNA expression. Significance of insulin-stimulated ANGPTL3/8 and ANGPTL4/8 secretion from hepatocytes was calculated using a two-tailed parametric paired *t*-test. Significance for BIL effects on circulating ANGPTL3/8 and ANGPTL4/8 complex levels was assessed using a two-way ANOVA with Dunnett’s multiple comparisons test.

## RESULTS

### Characterization of ANGPTL complexes

**Fig. 1.**
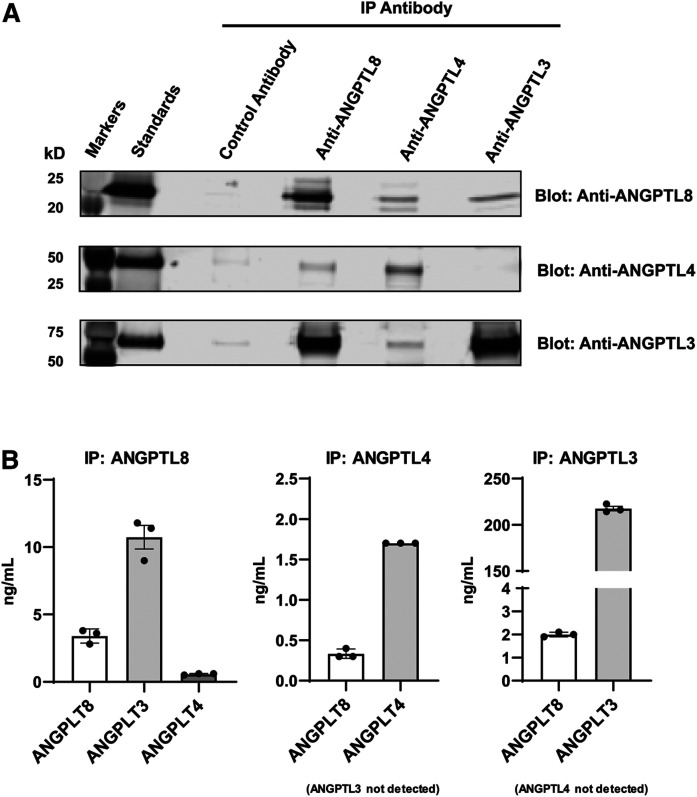
ANGPTL8 circulates in ANGPTL3/8 and ANGPTL4/8 complexes. A: Anti-ANGPTL8, anti-ANGPTL4, and anti-ANGPTL3 antibodies covalently coupled to beads, with heavy and light chains further cross-linked, were used to immunoprecipitate (IP) human serum. Proteins were separated on a 12% Bis-Tris gel and transferred to PVDF. Co-immunoprecipitating proteins were visualized via Western blotting. Results are representative of two independent experiments. B: ANGPTL8, ANGPTL4, and ANGPTL3 were immunoprecipitated from human serum. Beads were washed using PBS, and bound proteins were reduced with DTT and alkylated. Following digestion, digests were acidified, and co-immunoprecipitating proteins were quantified using a mass spectrometry LC-MRM method. Results are shown as the mean ± SEM (n = 3) from one experiment representative of two independent experiments.

**Fig. 2.**
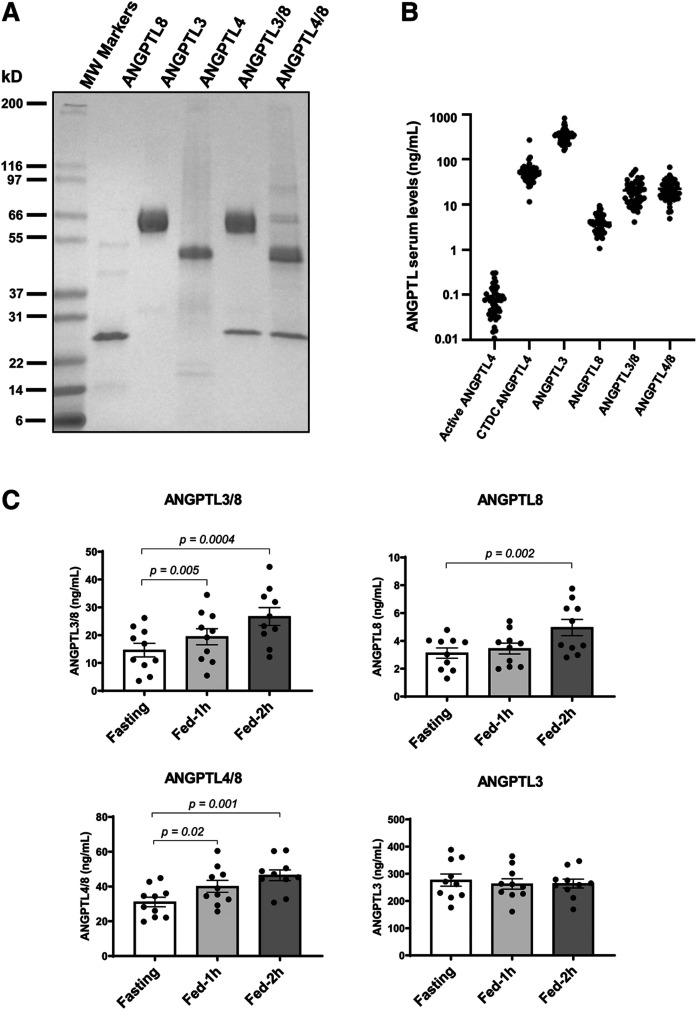
ANGPTL3/8 and ANGPTL4/8 complexes increase with feeding. A: Recombinant human ANGPTL proteins and complexes used for immunoassays were characterized via electrophoresis. One microgram of each recombinant protein or complex was analyzed using gradient gel electrophoresis with a 4–20% Tris-glycine gel, followed by Coomassie Blue staining. B: Active ANGPTL4 (defined as full-length ANGPTL4 or the N-terminal fragment of ANGPTL4), CTDC ANGPTL4, ANGPTL3, ANGPTL8, ANGPTL3/8, and ANGPTL4/8 were measured in 50 normal donors using dedicated sandwich immunoassays. C: ANGPTL3/8, ANGPTL4/8, ANGPTL3, and ANGPTL8 were measured using dedicated sandwich immunoassays in 10 normal donors during fasting conditions and 1 and 2 h following a mixed meal challenge. Results are shown as the mean ± SEM. Significance for the feeding effect on ANGPTL proteins and complexes was assessed using a paired *t*-test.

Based on reports that ANGPTL8 may interact with other ANGPTL proteins (39, 40, 49, 83), we immunoprecipitated ANGPTL8, ANGPTL3, and ANGPTL4 (using an N-terminal ANGPTL4 antibody) from human serum and identified co-immunoprecipitating proteins via Western blotting. As **Fig. 1A** shows, ANGPTL3 and ANGPTL4 did not co-immunoprecipitate with each other, while each co-immunoprecipitated with ANGPLT8, indicating the presence of ANGPTL3/8 and ANGPTL4/8 complexes. The existence of these complexes was confirmed via mass spectrometry. As Fig. 1B demonstrates, the amount of ANGPTL8 present in the ANGPTL3/8 and ANGPTL4/8 complexes was similar to the total amount of ANGPTL8 observed, suggesting that most ANGPTL8 in serum was present in either ANGPTL3/8 or ANGPTL4/8 complexes. While human serum contained ANGPTL3 at roughly 200 ng/ml, much less N-terminally intact (active) ANGPTL4 was present. Other than N-terminally intact ANGPTL4 present in ANGPTL4/8 complex, very little noncomplexed (free) active ANGPTL4 was observed. The main free circulating form of ANGPTL4 was subsequently determined by separate immunoassay experiments (**Fig. 2B**) to be CTDC ANGPTL4.

We also utilized a mass spectrometry LC-MRM method with SIL peptides to ascertain the molar ratios of the respective proteins in the recombinant ANGPTL3/8 and ANGPTL4/8 complexes. The protein ratios in the ANGPTL3/8 and ANGPTL4/8 complexes were found to be 3:1 and 1:1, respectively (**Table 1**). In addition, endogenous ANGPTL3/8 and ANGPTL4/8 complexes were immunoprecipitated from human serum and similarly characterized. The protein ratios in the endogenous ANGPTL3/8 and ANGPTL4/8 complexes were also found to be 3:1 and 1:1, respectively (Table 1), consistent with the ratios for the recombinant complexes.

**TABLE 1.** Determination of ANGPTL3/8 and ANGPTL4/8 protein ratios by mass spectrometry

Sample	Molar Ratio of ANGPTL3 Compared with Labeled Peptides			Molar Ratio of ANGPTL4 Compared with Labeled Peptides			Molar Ratio of ANGPTL8 Compared with Labeled Peptides			Protein Ratio for Each Respective Complex	
	Peptide #1	Peptide #2	Average	Peptide #1	Peptide #2	Average	Peptide #1	Peptide #2	Average	ANGPTL3/8	ANGPTL4/8
Recombinant complexes											
ANGPTL3/8											
Sample #1	9.60	12.96	11.28	—	—	—	3.64	2.92	3.28	3.4	—
Sample #2	9.19	13.74	11.47	—	—	—	3.64	3.04	3.34	3.4	—
Sample #3	8.06	13.45	10.75	—	—	—	3.23	2.92	3.08	3.5	—
SD	0.80	0.40	0.37	—	—	—	0.24	0.07	0.14	0.03	—
ANGPTL4/8											
Sample #1	—	—	—	0.71	0.81	0.76	0.88	0.74	0.81	—	0.9
Sample #2	—	—	—	0.54	0.67	0.60	0.68	0.57	0.62	—	1.0
Sample #3	—	—	—	0.68	0.92	0.80	0.74	0.62	0.68	—	1.2
SD	—	—	—	0.09	0.13	0.11	0.11	0.09	0.10	—	0.13
Endogenous complexes											
ANGPTL3/8											
Sample #1	1.27	1.34	1.31	**—**	**—**	**—**	0.37	0.38	0.37	3.5	**—**
Sample #2	1.25	1.27	1.26	—	—	—	0.37	0.36	0.36	3.4	—
SD	0.02	0.05	0.03	—	—	—	0.004	0.01	0.01	0.08	—
ANGPTL4/8											
Sample #1	—	—	—	0.041	0.055	0.048	0.062	0.058	0.060	—	0.8
Sample #2	—	—	—	0.043	0.052	0.048	0.059	0.062	0.061	—	0.8
SD	—	—	—	0.002	0.002	0.00	0.002	0.003	0.01	—	0.01

ANGPTL3/8 and ANGPTL4/8 complexes were digested using trypsin and Lys-C. Identical molar amounts of SIL peptides were spiked into samples during digestion, and the ratio of unlabeled to labeled peptides was determined. Stoichiometries of protein complexes were determined by comparing the averaged ratios derived from two peptides per protein. Data for recombinant complexes were derived from a single preparation performed in triplicate. Data for endogenous complexes were derived from a serum pool from 20 healthy donors and performed in duplicate. SDs are shown for each peptide in the technical replicates and for the protein ratios in the respective complexes.

### Measurement of ANGPTL proteins and complexes in human serum

We used recombinant ANGPTL proteins and complexes (**Fig. 2A**) to develop dedicated immunoassays to measure human serum levels of ANGPTL3, ANGPTL4, ANGPTL8, ANGPTL3/8 complex, and ANGPTL4/8 complex. For ANGPTL4, an assay using two N-terminal ANGPTL4 antibodies enabled measurement of full-length ANGPTL4 and N-terminal ANGPTL4 fragment (collectively referred to as active ANGPTL4). Likewise, an assay using C-terminal ANGPTL4 antibodies enabled measurement of full-length ANGPTL4 and (inactive) ANGPTL4 C-terminal fragment, collectively referred to as CTDC ANGPTL4. As Fig. 2B shows, active ANGPTL4 levels of roughly 0.1 ng/ml were more than three log orders lower than the ANGPTL3 concentrations and more than two log orders lower than those for CTDC ANGPTL4. Because levels of ANGPTL4 measured by this assay were so much less than ANGPTL4/8 levels, this assay likely did not detect ANGPTL4 present in ANGPTL4/8 complexes to any appreciable extent. These data confirmed our mass spectrometry-based observations and indicated that most free circulating ANGPTL4 consisted of inactive C-terminal fragment, a concept consistent with our subsequent LPL activity data. Concentrations of ANGPTL8, ANGPTL3/8, and ANGPTL4/8, averaged 4 ng/ml, 20 ng/ml, and 23 ng/ml, respectively.

Overall, the protein concentrations obtained using our immunoassays compared reasonably well to the mass spectrometry-based estimates, especially considering the multiple steps required for mass spectrometry assessments. Levels of each of the respective proteins and complexes were also compared with serum TG concentrations. Interestingly, only ANGPTL8, ANGPTL3/8, and ANGPTL4/8 were significantly positively correlated with circulating TG (*R*-values of 0.47, 0.51, and 0.36, respectively, and *P*-values of 0.0007, 0.0002, and 0.01, respectively). There was no significant correlation of ANGPTL3, active ANGPTL4, or CTDC ANGPTL4 with serum TG.

Based on these findings, we used our immunoassays to measure levels of ANGPTL3, ANGPTL8, ANGPTL3/8, and ANGPTL4/8 in serum samples collected from normal subjects while fasting and 1 and 2 h following a mixed meal challenge. As shown in Fig. 2C, ANGPTL3 concentrations did not change meaningfully in the postprandial state. In contrast, ANGPTL3/8 and ANGPTL4/8 both increased significantly postprandially, consistent with increases observed in ANGPTL8.

We further explored circulating ANGPTL3/8 and ANGPTL4/8 levels by measuring these complexes in 352 control subjects from the SCARF cardiovascular outcomes study (84–86). The average ANGPTL3/8 level in the SCARF samples was 17 ng/ml, while the average ANGPTL4/8 level was 23 ng/ml. These levels were similar to those observed in the previously studied healthy subjects. As **Table 2** shows, circulating ANGPTL3/8 and ANGPTL4/8 levels were inversely correlated with HDL-C and directly correlated with TG, fasting glucose, fasting insulin, waist to hip ratio, and BMI, as well as systolic and diastolic blood pressure. In addition, ANGPTL3/8 concentrations (but not ANGPTL4/8 concentrations) were also positively correlated with total cholesterol and LDL-C. Finally, ANGPTL3/8 and ANGPTL4/8 were also directly correlated with each other (*R* = 0.39, *P* < 0.0001).

**TABLE 2.** Circulating ANGPTL3/8 and ANGPTL4/8 are highly correlated with metabolic syndrome markers

Phenotype	ANGPTL3/8		ANGPTL4/8	
	*R*-Value	*P*	*R*-Value	*P*
TG	0.485	<0.0001	0.261	<0.0001
HDL-C	−0.279	<0.0001	−0.247	<0.0001
LDL-C	0.218	<0.0001	0.062	0.24 (NS)
Total cholesterol	0.233	<0.0001	0.072	0.18 (NS)
BMI	0.484	<0.0001	0.377	<0.0001
Waist-hip ratio	0.351	<0.0001	0.240	<0.0001
Fasting glucose	0.282	<0.0001	0.215	<0.0001
Fasting insulin	0.635	<0.0001	0.473	<0.0001
Systolic blood pressure	0.187	0.0005	0.196	0.0003
Diastolic blood pressure	0.286	0.0002	0.234	<0.0001

ANGPTL3/8 and ANGPTL4/8 complexes were measured in fasting serum samples from SCARF subjects (n = 352), and their associations with various metabolic parameters were assessed.

### ANGPTL3/8 inhibition of LPL-facilitated hepatic VLDL-C uptake

**Fig. 3.**
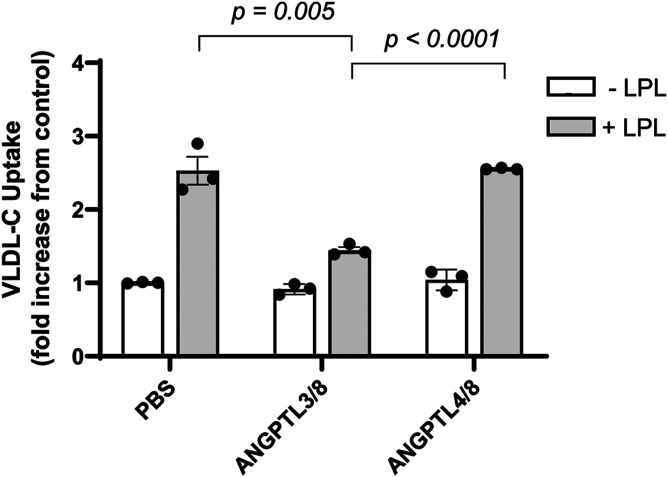
ANGPTL3/8 blocks LPL-facilitated hepatocyte VLDL-C uptake. Cholesterol uptake in Huh7 hepatocytes was measured in the absence or presence of LPL pre-incubated with vehicle, ANGPTL3/8 complex, or ANGPTL4/8 complex for 1 h before mixing with fluorescent-labeled VLDL, followed by addition to the Huh7 hepatocytes for 30 min. The media was then replaced with fixative. Cells were fixed for 20 min, washed twice with PBS, and covered with PBS. Fluorescence at 495/525 nm was measured, with VLDL uptake calculated as relative fluorescent units at 525 nm. Results are shown as the mean ± SEM (n = 3).

After analyzing the SCARF samples and noting that ANGPTL3/8 was positively correlated with LDL-C, we turned our attention toward understanding why this might be the case. In so doing, we examined the ability of ANGPTL3/8 to affect LPL-facilitated hepatocyte VLDL-C uptake. We chose ANGPTL4/8 as the control for these experiments after observing that: *1*) ANGPTL3 showed no correlation with TG in clinical samples; *2*) serum levels of active ANGPTL4 were negligible compared with those of ANGPTL3/8 and ANGPTL4/8; and *3*) we could not detect any effect of ANGPTL8 alone on LPL activity. As **Fig. 3** shows, addition of LPL to VLDL-C-containing media significantly increased Huh7 hepatocyte uptake of VLDL-C, consistent with previous reports (54, 87, 91, 92). When ANGPTL3/8 was pre-incubated with LPL prior to addition of LPL to the media, however, hepatocyte VLDL-C uptake was reduced nearly to levels observed in the absence of LPL. In contrast, when ANGPTL4/8 was pre-incubated with LPL prior to addition of LPL to the media, there was no significant effect on LPL-facilitated VLDL-C uptake by the hepatocytes. Together, these results suggested that ANGPTL3/8 may inhibit the ability of LPL to facilitate hepatic uptake of cholesterol-containing lipoproteins. An important caveat in interpreting these data, however, is that the results reflect VLDL particle uptake by multiple different receptors (including LDLR and VLDLR), with several required steps (including lipolysis, attachment, and internalization). Therefore, reduced uptake in this assay could be due to both direct effects on particle uptake as well as indirect effects on VLDL clearance mechanisms.

### Binding of ANGPTL complexes to LPL

**Fig. 4.**
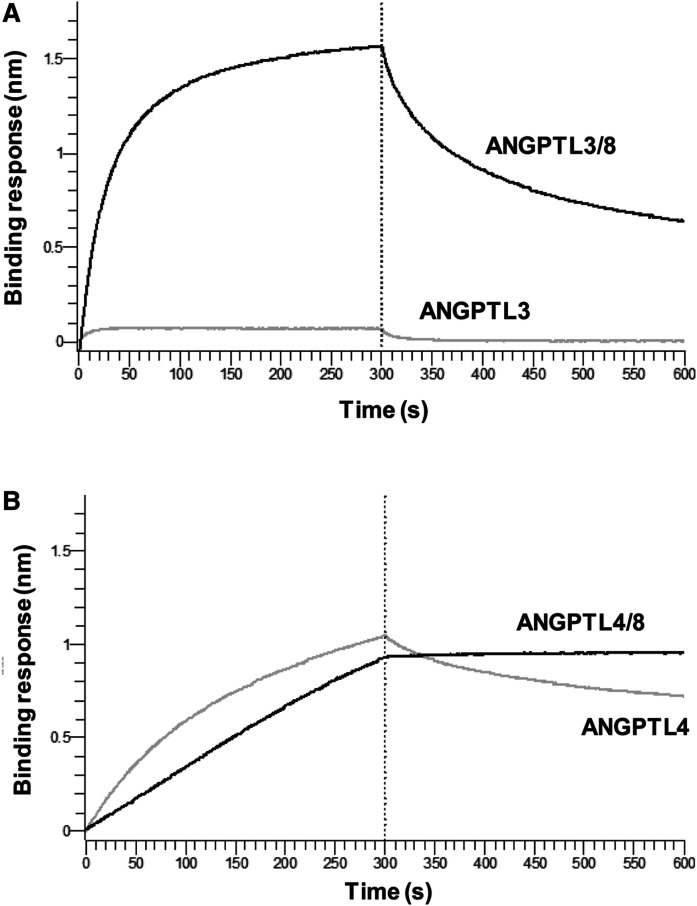
ANGPTL3/8 and ANGPTL4/8 manifest different binding patterns to LPL. A: The ability of ANGPTL3 and ANGPTL3/8 to bind LPL was assessed with bio-layer interferometry. Avidin-tagged LPL was immobilized on streptavidin biosensors and incubated with ANGPTL3 or ANGPTL3/8 and transferred to buffer-only wells to monitor dissociation. The left side of the graph shows the association of ANGPTL3 and ANGPTL3/8 with LPL. The right side shows their respective dissociations. Results are representative of three independent experiments. B: The ability of ANGPTL4 and ANGPTL4/8 to bind LPL was assessed with bio-layer interferometry. Avidin-tagged LPL was immobilized on streptavidin biosensors and incubated with ANGPTL4 or ANGPTL4/8 and transferred to buffer-only wells to monitor dissociation. The left side of the graph shows the association of ANGPTL4 and ANGPTL4/8 with LPL. The right side shows their respective dissociations. Results are representative of three independent experiments.

To better understand the potential differences in LPL interactions with ANGPTL3/8 and ANGPTL4/8, we examined the in vitro binding of purified ANGPTL3, ANGPTL4, ANGPTL3/8, and ANGPTL4/8 to LPL using bio-layer interferometry. As **Fig. 4A** shows, ANGPTL3 demonstrated weak binding to LPL, whereas ANGPTL3/8 demonstrated markedly increased binding, similar to that observed with ANGPTL4 alone (Fig. 4B). In contrast, ANGPTL4/8 demonstrated a very different binding pattern, with a much slower off-rate than what was observed for either ANGPTL3/8 or ANGPTL4. **Table 3** shows a summary of the LPL-binding kinetics for ANGPTL3, ANGPTL3/8, ANGPTL4, and ANGPTL4/8.

**TABLE 3.** LPL-binding characteristics of ANGPTL proteins and complexes

ANGPTL Protein or Complex	K_d_ (nM)	K_on_ (1/Ms)	K_off_ (1/s)
ANGPTL3	343.0	3.2 x 10^4^	1.2 × 10^−3^
ANGPTL3/8	6.4	6.9 x 10^4^	4.4 × 10^−3^
ANGPTL4	17.7	9.7 x 10^5^	1.7 × 10^−3^
ANGPTL4/8	<0.001	4.8 x 10^4^	<1 × 10^−7^

The K_d_, K_on_, and K_off_ for ANGPTL3, ANGPTL3/8, ANGPTL4, and ANGPTL4/8 binding to LPL were determined using bio-layer interferometry.

### Effect of ANGPTL3, ANGPTL3/8, ANGPTL4, and ANGPTL4/8 on LPL activity

**Fig. 5.**
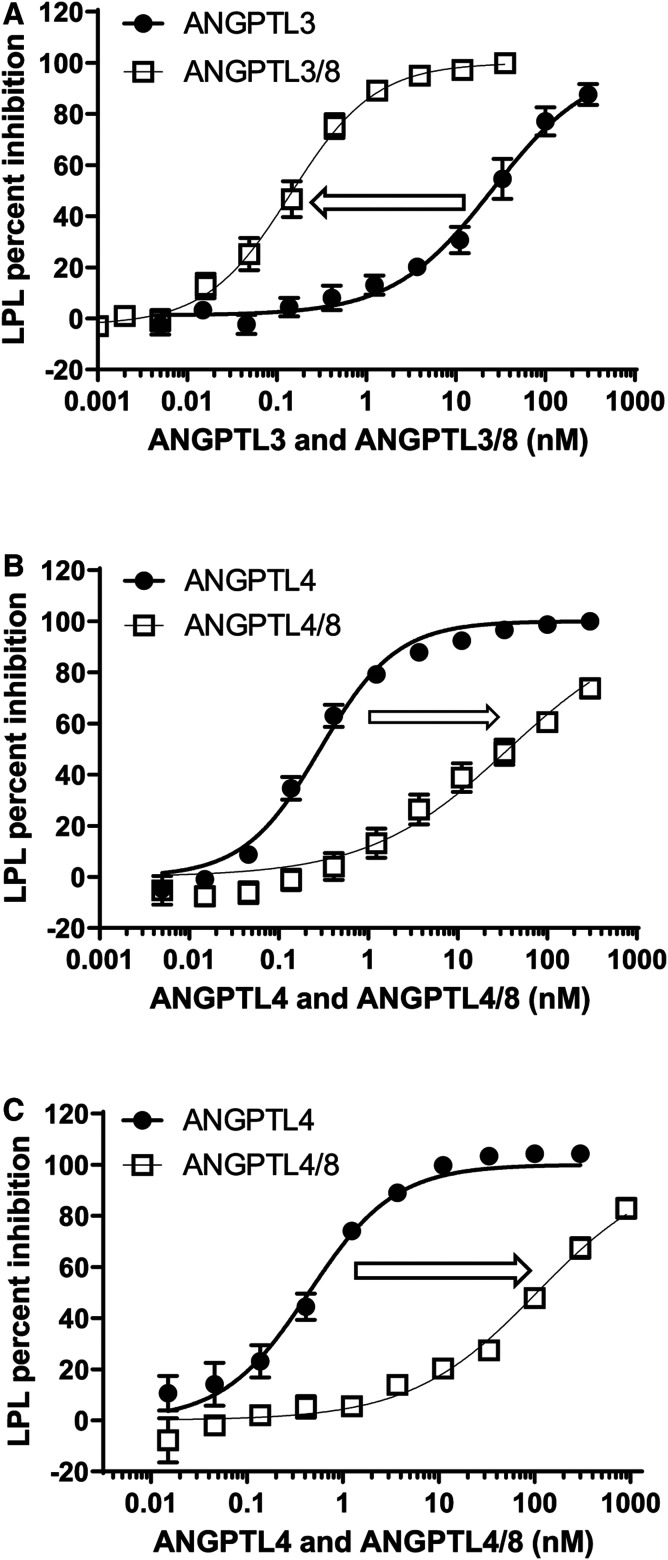
ANGPTL8 markedly increases ANGPTL3 inhibition of LPL but dramatically decreases ANGPTL4 inhibition of LPL. A: The ability of ANGPTL3 or ANGPTL3/8 to inhibit LPL was assessed using LPL-stable expression cells incubated with ANGPTL3 or ANGPTL3/8 prior to the addition of lipase substrate. Fluorescence was monitored at 1 and 30 min to correct for background. ANGPTL3/8 showed a 186-fold increase in LPL inhibition compared to ANGPTL3 alone (IC_50_ values of 0.14 nM versus 26 nM, respectively). Results are shown as the mean ± SEM (n = 5). B: The ability of ANGPTL4 or ANGPTL4/8 to inhibit LPL was similarly assessed. ANGPTL4/8 showed a 128-fold decrease in LPL inhibition compared to ANGPTL4 alone (IC_50_ values of 37 nM versus 0.29 nM, respectively). Results are shown as the mean ± SEM (n = 6). C: ANGPTL4 or ANGPTL4/8 inhibition of LPL was assessed using VLDL as a substrate. The assay was similar to that used in A and B, except that lipase substrate was replaced with VLDL and free FAs were measured. ANGPTL4/8 showed a 239-fold decrease in LPL inhibition compared to ANGPTL4 alone (IC_50_ values of 105 nM versus 0.44 nM, respectively). Results are shown as the mean ± SEM (n = 3).

We next examined the effect that ANGPTL8 had on the ability of ANGPTL3 to inhibit LPL enzymatic activity (53, 59). On its own, ANGPTL3 demonstrated inhibition of LPL with an IC_50_ of 26 nM (**Fig. 5A**). When ANGPTL8 was present together with ANGPTL3 in an ANGPTL3/8 complex, however, the inhibition increased markedly, with ANGPTL3/8 demonstrating a 186-fold increase in potency compared with ANGPTL3 alone (IC_50_ of 0.14 nM). ANGPTL4 was then evaluated in the LPL activity assay (Fig. 5B) and demonstrated inhibition with an IC_50_ of 0.29 nM (similar to that observed for ANGPTL3/8). In stark contrast to the results obtained with ANGPTL3, when ANGPTL8 was combined with ANGPTL4 to form ANGPTL4/8 complex, potency was reduced 128-fold (IC_50_ of 37 nM), indicating that ANGPTL8 was drastically decreasing the ability of ANGPTL4 to inhibit LPL. In light of the marked decrease in LPL inhibitory activity of ANGPTL4/8 versus ANGPTL4, we performed an additional VLDL substrate-based activity assay to confirm this result (Fig. 5C). In this assay, ANGPTL4/8 showed a 239-fold decrease in potency of LPL inhibition compared with ANGPTL4 alone (IC_50_ of 105 nM versus 0.44 nM, respectively). **Table 4** summarizes these results.

**TABLE 4.** LPL inhibition summary for ANGPTL3 versus ANGPTL3/8 and ANGPTL4 versus ANGPTL4/8 activity assays

Enzyme	Substrate	ANGPTL Protein	IC_50_ (nM)	ANGPTL Complex	IC_50_ (nM)	Change Direction	Fold Change
LPL	BODIPY-TG	ANGPTL3	26	ANGPTL3/8	0.14	Increase	(+) 186
LPL	BODIPY-TG	ANGPTL4	0.29	ANGPTL4/8	37	Decrease	(−) 128
LPL	VLDL	ANGPTL4	0.44	ANGPTL4/8	105	Decrease	(−) 239

IC_50_ concentrations were determined for ANGPTL3 versus ANGPTL3/8 and ANGPTL4 versus ANGPL4/8 in LPL.

### ANGPTL4/8 blocking of ANGPTL3/8- and ANGPTL4-mediated inhibition of LPL

**Fig. 6.**
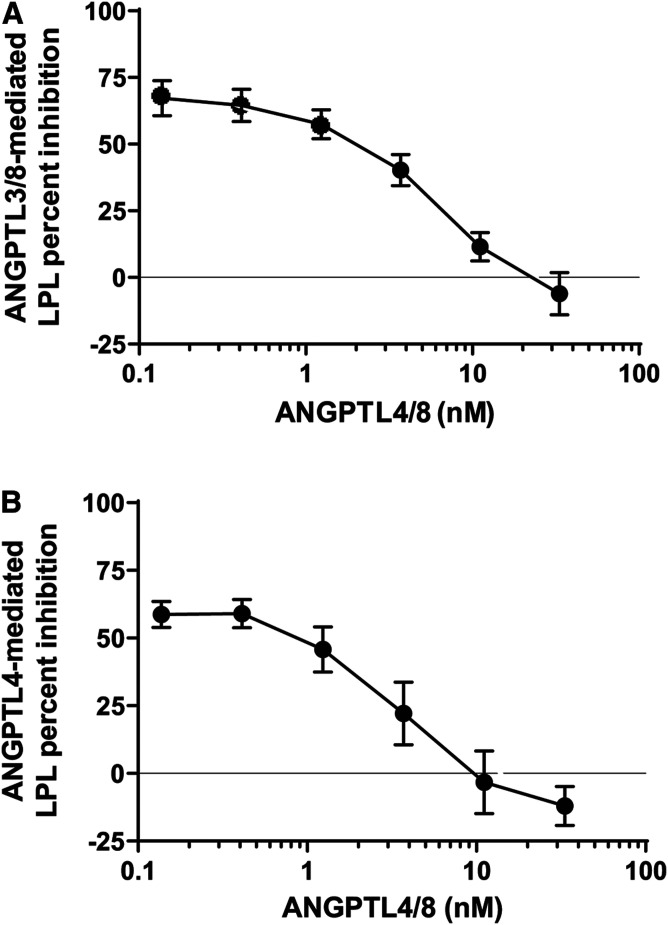
ANGPTL4/8 blocks ANGPTL3/8- and ANGPTL4-mediated inhibition of LPL. A: To study the ability of ANGPTL4/8 to protect LPL from ANGPTL3/8 inhibition, various concentrations of ANGPTL4/8 were pre-incubated with LPL-stable expression cells for 1 h. Afterward, 1 nM of ANGPTL3/8 was added for a further 1 h incubation, prior to the addition of lipase substrate. Fluorescence was monitored as in Fig. 5A. Results are shown as the mean ± SEM (n = 4). B: To study the ability of ANGPTL4/8 to protect LPL from ANGPTL4 inhibition, various concentrations of ANGPTL4/8 were pre-incubated with LPL-stable expression cells for 1 h. Afterward, 1 nM of ANGPTL4 was added for a further 1 h incubation, prior to the addition of lipase substrate. Fluorescence was monitored as in Fig. 5A. Results are shown as the mean ± SEM (n = 6).

The studies presented in Fig. 5 indicated that binding of ANGPTL8 to ANGPTL3 markedly enhanced the inhibitory effect of ANGPTL3 on LPL activity. In contrast, while ANGPTL4 alone had an inhibitory effect comparable to that of the ANGPTL3/8 complex on LPL activity, the binding of ANGPTL8 to ANGPTL4 markedly reduced this inhibitory effect of ANGPTL4 on LPL activity. These data suggested that when bound to LPL, the ANGPTL4/8 complex might also act as a “bodyguard” to protect LPL from the inhibitory effect of the ANGPTL3/8 complex. We thus hypothesized that the tight binding of the ANGPTL4/8 complex to LPL and its slow dissociation rate might prevent LPL inhibition by ANGPTL3/8. This prompted us to assess the ability of ANGPTL3/8 to inhibit LPL activity after pre-incubation of LPL with ANGPTL4/8. In these experiments, increasing amounts of ANGPTL4/8 proportionally decreased the ability of ANGPTL3/8 to inhibit LPL (**Fig. 6A**).

After obtaining these results, we performed experiments to determine whether increasing amounts of ANGPTL4/8 could also decrease the ability of ANGPTL4 to inhibit LPL. As shown in Fig. 6B, this proved to be the case. Together, these results indicated that ANGPTL4/8 can effectively compete with both ANGPTL3/8 and ANGPTL4 for binding to LPL and, in so doing, block the ability of ANGPTL3/8 and ANGPTL4 to inhibit LPL. In these experiments, pre-incubation of LPL with 10 nM of ANGPTL4/8 was required to completely block the inhibition of LPL by 1 nM ANGPTL3/8 (and 1 nM ANGPTL4), indicating that high local concentrations of ANGPTL4/8 may be required to prevent circulating ANGPTL3/8 from inhibiting LPL in the fat. These observations suggested a mechanism by which ANGPTL4/8 localized in adipose tissue could block circulating ANGPTL3/8 from inhibiting LPL in the fat, thus ensuring that increased ANGPTL3/8 after feeding inhibits LPL mainly in skeletal muscle. This concept is consistent with the serum levels observed for ANGPTL3/8 and ANGPTL4/8. ANGPTL3/8 would be expected to act in an endocrine manner, as its serum level falls midway on its LPL inhibition curve. In contrast, circulating ANGPTL4/8 levels are far lower than those required to block the ability of circulating ANGPTL3/8 to inhibit LPL, consistent with ANGPTL4/8 acting more in an autocrine/paracrine manner.

### Insulin-stimulated release of ANGPTL3/8 from hepatocytes

**Fig. 7.**
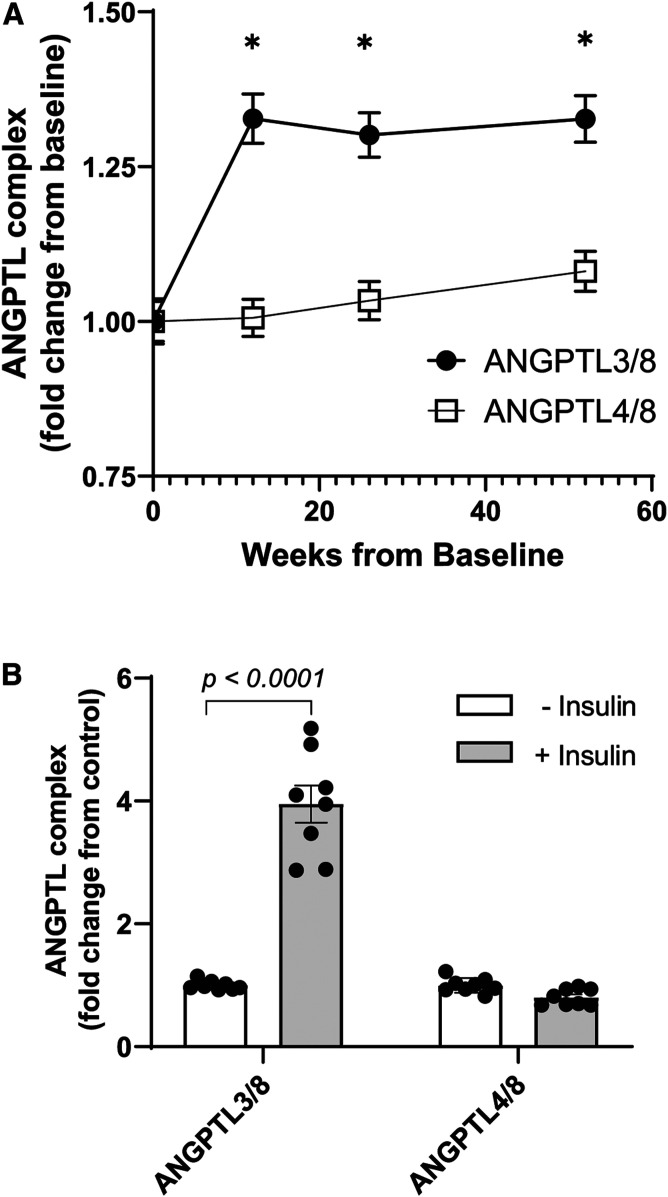
Insulin stimulates human hepatocyte secretion of ANGPTL3/8. A: Insulin-naïve patients (n = 279) were administered the hepatic-preferential insulin BIL, and serum samples were obtained under morning fasting conditions over the course of 1 year of BIL treatment. ANGPTL3/8 and ANGPTL4/8 levels were measured at baseline and after 12, 26, and 52 weeks of BIL administration. Results are shown as the mean ± SEM (*P < 0.0001 versus week 0). B: Human primary hepatocytes obtained in the HepatoPac platform were washed in serum-free application media and pre-incubated in application media in the absence of insulin. Following aspiration, cells were incubated with application media in the absence or presence of 1 nM of insulin. ANGPTL3/8 and ANGPTL4/8 levels in the media were measured using sandwich immunoassays, with the results shown as the mean ± SEM (n = 8).

We next turned our attention to the source of increased postprandial ANGPTL3/8 that we observed in human serum. Based on previous reports that hepatic ANGPTL8 mRNA increases in the fed state, we hypothesized that insulin might stimulate the secretion of ANGPTL3/8 from the liver (41). To test this hypothesis, we measured ANGPTL3/8 at multiple time points in insulin-naïve patients treated for 52 weeks with BIL, a hepato-preferential insulin (89, 90). Mean baseline ANGPTL3/8 and ANGPTL4/8 levels in these type 2 diabetes patients were 19 and 45 ng/ml, respectively. As **Fig. 7A** shows, the hepato-selective insulin significantly increased ANGPTL3/8 circulating concentrations. In comparison, there was little change in circulating ANGPTL4/8 levels, suggesting that the source of increased postprandial ANGPTL4/8 was not the liver. To confirm further that insulin stimulated the release of ANGPTL3/8 (but not ANGPTL4/8) from hepatocytes, we also incubated human primary hepatocytes in the absence or presence of 1 nM insulin and measured levels of secreted ANGPTL3/8 and ANGPTL4/8 complexes. As Fig. 7B shows, insulin stimulation significantly increased hepatocyte release of ANGTL3/8, while not affecting the release of ANGPTL4/8. Together with the in vivo BIL data, these results confirmed that insulin stimulates hepatic secretion of ANGPTL3/8.

### Insulin-stimulated release of ANGPTL4/8 from adipocytes

The above observations indicated that insulin did not increase ANGPTL4/8 release from the liver and caused us to hypothesize that insulin-stimulated secretion of ANGPTL4/8 might occur from the fat. We considered previous reports describing that while ANGPTL8 mRNA was highly insulin-responsive in human adipocytes, levels of the secreted protein did not increase upon insulin treatment (93, 94). We hypothesized that there might be two reasons for this. The first could be that a further confounding factor might prevent the ability of adipocytes to release the ANGPTL4/8 complex in vitro. The second could be that another signal in addition to insulin might be required for maximal ANGPTL4/8 secretion from adipocytes. In this latter scenario, in order for postprandial increases in ANGPTL4/8 to occur, increased insulin levels alone might not be sufficient, but rather an additional stimulus might be required. If a second signal beyond insulin was indeed required for optimal ANGPTL4/8 secretion from adipose tissue, we believed a likely candidate would be GIP, an incretin secreted by K-cells in the gut in response to fat and carbohydrate intake, as the GIP receptor is highly expressed in adipocytes (95). Further influencing our thinking was the fact that we had shown in a previous study that postprandial increases in GIP manifested a pattern very similar to those observed for the postprandial ANGPTL4/8 increases observed in our current study (95).

**Fig. 8.**
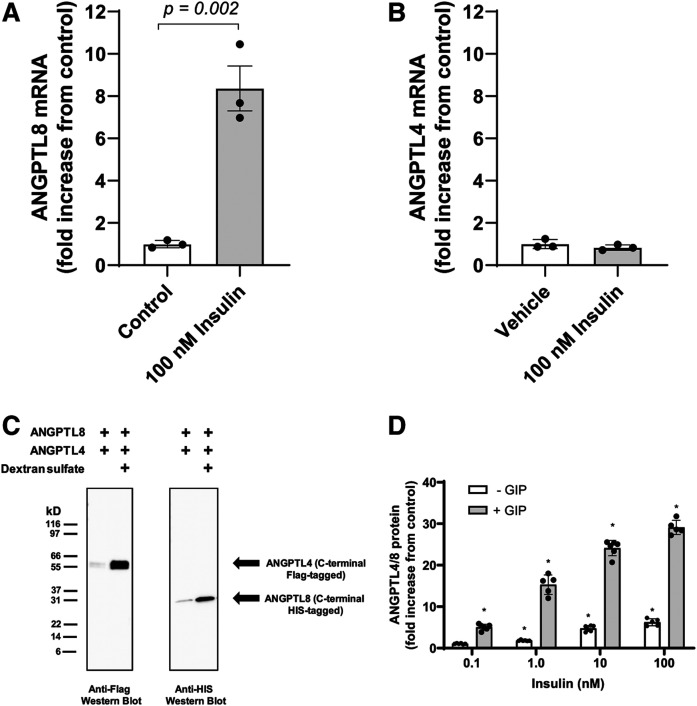
Insulin stimulates ANGPTL4/8 secretion from human adipocytes. A: Human adipocytes were incubated in the absence or presence of insulin, and 1 μg of total RNA was reverse transcribed. ANGPTL8 transcript levels were quantitated. Insulin treatment resulted in an approximate 8-fold increase in ANGPTL8 mRNA levels. Results are shown as the mean ± SEM (n = 3). B: ANGPTL4 transcript levels were quantitated in the human adipocytes in A. Results are shown as the mean ± SEM (n = 3). C: Flag-tagged ANGPTL4 and HIS-tagged ANGPTL8 constructs were transfected into HEK293 cells. Afterward, dextran sulfate was added, media were harvested, and equal volumes from each condition were immunoblotted with anti-Flag or anti-HIS antibody. D: Human adipocytes were treated in heparin-containing media supplemented with 0–100 nM insulin in the absence and presence of 10 nM GIP. Media were collected and analyzed for ANGPTL4/8. Results are shown as the mean ± SEM (n = 6, *P < 0.0001 versus control).

We therefore turned our attention to measuring insulin-stimulated secretion of ANGPTL4/8 from adipocytes. As shown in **Fig. 8A**, we confirmed that exposure of human adipocytes to insulin increased levels of ANGPTL8 mRNA. In contrast, ANGPTL4 mRNA levels did not change with insulin treatment (Fig. 8B). We also observed that ANGPTL3 mRNA levels were undetectable, consistent with previous reports that adipocytes do not express ANGPTL3 (96). To understand why previous researchers were unable to measure insulin stimulated ANGPTL8 release from adipocytes, we considered that ANGPTL8 might be secreted as part of an ANGPTL4/8 complex that could remain tightly bound to plasma membranes via interaction with heparin sulfate proteoglycans, thereby preventing release into the media. To understand whether this might be the case, we first expressed ANGPTL8 and ANGPTL4 in HEK293 cells in the absence or presence of 0.1 mg/ml dextran sulfate (a heparin-like compound). Interestingly, the addition of dextran sulfate greatly increased the release of ANGPTL4 and ANGPTL8 (Fig. 8C), causing us to conduct subsequent experiments in adipocytes in the presence of heparin.

We thus treated adipocytes in heparin-containing media with insulin in the absence or presence of GIP and measured release of ANGPTL4/8. Under these conditions, insulin dose-dependently increased the secretion of ANGPTL4/8, and this dose-dependent increase was greatly augmented by the addition of GIP (Fig. 8D). In the absence of GIP, 1, 10, and 100 nM insulin increased adipocyte ANGPTL4/8 secretion by 2.1-, 6.5-, and 7.7-fold, respectively, compared with control, whereas in the presence of GIP, 1, 10, and 100 nM of insulin increased adipocyte ANGPTL4/8 secretion by 14.4-, 22.7-, and 27.4-fold, respectively.

In contrast, no ANGPTL4/8 was measurable in the absence of heparin, indicating that ANGPTL4/8 secreted by adipocytes may remain mostly localized in the adipose tissue in vivo. This suggests that ANGPTL4/8 present in the circulation likely reflects adipose tissue ANGPTL4/8 concentrations, with ANGPTL4/8 entering the circulation from blood flow through the adipose capillary beds. Of note, we also attempted to measure ANGPTL3/8 secreted from the adipocytes stimulated with insulin but were unable to detect any ANGPTL3/8 in the media, consistent with undetectable adipocyte ANGPTL3 mRNA levels.

Together, these results indicated that by staying mainly localized in the adipose tissue, increased postprandial ANGPTL4/8 may prevent the increased postprandial circulating ANGPTL3/8 (as well as localized ANGPTL4) from inhibiting LPL in the fat. This suggests a mechanism for the elevated circulating ANGPTL3/8 that occurs after feeding to act mainly in the skeletal muscle and not the adipose tissue, thereby ensuring that conflicting LPL inhibitory signals are not sent to both tissues simultaneously.

## DISCUSSION

In this study, we show that ANGPTL8 is an insulin-responsive mediator of FA uptake that directs the storage of calories from food into the fat for future energy needs. ANGPTL8 does this by forming ANGPTL3/8 and ANGPTL4/8 complexes with respective protein ratios of 3:1 and 1:1. By forming an ANGPTL3/8 complex, ANGPTL8 markedly increases ANGPTL3 inhibition of LPL to decrease skeletal muscle LPL activity and thus decrease skeletal muscle FA uptake. Through forming an ANGPTL4/8 complex, ANGPTL8 markedly decreases ANGPTL4 inhibition of LPL to increase LPL activity in the fat to facilitate adipose tissue FA uptake. Through its tight binding to adipocyte-associated LPL, ANGPTL4/8 may also block the ability of circulating ANGPTL3/8 (and localized ANGPTL4) to inhibit LPL in adipose tissue. These properties of ANGPTL8 allow for the postprandial increase of LPL-inhibitory activity of ANGPTL3/8 to occur mainly in the skeletal muscle so that FA are taken up mostly into the fat after feeding.

This system provides a mechanism to ensure that LPL in adipose tissue is active after feeding while LPL in muscle is inhibited, thus allowing for proper storage of dietary lipids and preventing ectopic fat deposition. ANGPTL4/8 present in the circulation probably comes from localized ANGPTL4/8 in the fat that becomes detached as a result of the capillary flow across luminal surfaces of the adipose endothelium. In our experiments in HEK293 cells and adipocytes, ANGPTL4/8 was only released in vitro in the presence of dextran sulfate or heparin. These in vitro models, however, are unable to mimic the dynamic capillary flow that may cause some localized ANGPTL4/8 to enter the circulation. In addition, we cannot rule out the possibility, that some ANGPTL4/8 (as well as some ANGPTL4) that we detected in the circulation might come from skeletal muscle where ANGPTL4 has also been shown to be expressed (97).

In our study, ANGPTL3/8 demonstrated more than a 100-fold increased potency of LPL inhibitory activity compared with ANGPTL3, while ANGPTL4/8 showed at least a 100-fold decreased potency of LPL-inhibitory activity compared with ANGPTL4. The changes in potency suggest that these proteins and their complexes exist in a symmetrically modifiable system. ANGPTL4 is a much more potent inhibitor of LPL than ANGPTL3. Formation of the ANGPTL4/8 complex greatly diminishes ANGPTL4’s LPL-inhibitory activity to the point that it becomes similar to that of ANGPTL3. In contrast, formation of the ANGPTL3/8 complex greatly increases ANGPTL3’s LPL-inhibitory activity, resulting in an LPL inhibition profile comparable to that of ANGPTL4. An important caveat, however, is that our in vitro functional experiments were performed in conditions bearing little resemblance to capillary endothelial surfaces, where LPL acts in vivo. This is potentially important because several proteins (including APOC2, APOC3, and GPIHBP1) can affect LPL activity and stability, and may modulate the effects of ANGPTL proteins and complexes (51–55). Nevertheless, our data showing that circulating levels of active (N-terminally intact) ANGPTL4 are negligible compared with ANGPTL3/8 are consistent with our in vitro functional observations. Because active ANGPTL4 inhibits LPL as potently as ANGPTL3/8, it would be difficult for the system to operate properly if both active ANGPTL4 and ANGPTL3/8 reached the skeletal muscle at comparable levels.

Our study is the first to examine in detail the circulating levels of ANGPTL3/8 and ANGPTL4/8 complexes in man. Our data are consistent with the idea that most, if not all, ANGPTL8 released from hepatocytes is secreted as part of the ANGPTL3/8 complex. In contrast, our data also indicate that while some ANGPTL3 is secreted by the liver as part of an ANGPTL3/8 complex, most ANGPTL3 is secreted as free ANGPTL3 not complexed with ANGPTL8. Interestingly, ANGPTL3/8 and ANGPTL4/8 circulate at similar levels (roughly 20 ng/ml). For ANGPTL3/8, these levels are close to the IC_50_ we observed for the ANGPTL3/8 complex on LPL activity, consistent with the idea that circulating ANGPTL3/8 works in an endocrine manner. In the case of ANGPTL4/8, however, these levels are well below those required for a direct effect on LPL activity or for blocking the LPL-inhibitory effects of ANGPTL4 and ANGPTL3/8, further supporting the idea that the ANGPTL4/8 complex acts mainly in an autocrine/paracrine manner.

In our competition experiments, pre-incubation of LPL with ANGPTL4/8 blocked the inhibition of LPL by ANGPTL3/8, indicating that localized ANGPTL4/8 may prevent circulating ANGPTL3/8 from inhibiting LPL in the fat. These results suggest a mechanism by which ANGPTL4/8 localized in adipose tissue could block circulating ANGPTL3/8 from inhibiting LPL in the fat, thus ensuring that increased ANGPTL3/8 after feeding inhibits LPL mainly in skeletal muscle. We also contemplated examining the effect of active ANGPTL4 on ANGPTL3/8-mediated LPL inhibition, however because active ANGPTL4 and ANGPTL3/8 inhibit LPL to almost the same extent, it would have been extremely difficult to sort out ANGPTL4-mediated inhibition of LPL versus that caused by ANGPTL3/8.

In our in vitro binding experiments, ANGPTL4 and ANGPTL3/8 had similar, relatively high off rates with regard to their LPL binding. In contrast, ANGPTL4/8 demonstrated a very low off rate. In spite of this, ANGPTL4/8 showed much less inhibition of LPL than did ANGPTL4, causing us to reflect on how ANGPTL4/8 could bind to LPL with comparable or higher affinity compared with ANGPTL4 but without inhibiting it to the same degree. It is possible that ANGPTL4 and ANGPTL4/8 might bind to different domains of LPL, with ANGPTL4 binding causing marked LPL inhibition while ANGPTL4/8 binds to a somewhat different domain, resulting in much less inhibitory effect. This concept is consistent with the idea that binding alone, even high affinity binding, does not necessarily guarantee inhibition. Understanding exactly why ANGPTL4/8 can bind LPL with comparable or higher affinity than ANGPTL4 binds LPL, but without inhibiting it to the same extent, will be an important area of future study.

Additional novel findings in our study are the inverse correlations of ANGPTL3/8 and ANGPTL4/8 with HDL, and the direct correlations of both complexes with all other metabolic syndrome markers. For ANGPTL3/8, the positive correlation with TG and other markers of metabolic syndrome was not surprising. In the case of ANGPTL4/8, however, it was less obvious why despite relieving ANGPTL4-mediated LPL inhibition, ANGPTL 4/8 was also positively correlated with serum TG. One possibility might be that by blocking ANGPTL3/8 inhibition of LPL in the fat, ANGPTL4/8 shifts more ANGPTL3/8 to the skeletal muscle, where it inhibits LPL activity and thus decreases FA uptake into skeletal muscle, resulting in increased circulating TG. Further experiments will be needed to test this idea. Interestingly, we noted that ANGPTL3/8 was directly correlated with LDL-C, while ANGPTL4/8 was not. This could be related to LPL-facilitated uptake of cholesterol-containing lipoprotein particles into the liver via VLDL and related receptors (87, 98–102). Our data demonstrating that ANGPTL3/8 inhibited LPL-facilitated hepatocyte VLDL-C uptake might provide a possible explanation for the positive correlation of ANGPTL3/8 with LDL-C, but further mechanistic investigations along these lines will be needed.

Our observations build upon those of Zhang (42) (who proposed an ANGPTL3-4-8 model), Kovrov et al. (83) (who incubated N-terminal ANGPTL3 and ANGPTL4 fragments with ANGPTL8 and showed approximate 4-fold activation of ANGPTL3 and 4-fold inhibition of ANGPTL4), and Chi et al. (39) (who showed that ANGPTL8 complexed with ANGPTL3 and enhanced its ability to bind and inhibit LPL). Importantly, the study by Chi et al. (39) also showed that ANGPTL8 required complex formation in order to be efficiently secreted. This helps to explain why, in our present study, almost all serum ANGPTL8 was observed in complexes with ANGPTL3 or ANGPTL4.

Our observations in this study are also consistent with the observed ANGPTL knockout phenotypes (74, 79, 103). Humans with ANGPTL3 knockout mutations have decreased TG and LDL-C and decreased cardiovascular risk (72). These mutations might reduce circulating ANGPTL3/8, resulting in increased LPL activity and uptake of FA into skeletal muscle, thus lowering TG levels. Decreased circulating ANGPTL3/8 complex might also result in less inhibition of LPL-mediated cholesterol uptake by the liver, thereby lowering LDL-C levels. In contemplating why an ANGPTL4 knockout or E40K mutation would be beneficial, one possibility might be that reduction of ANGPTL4 in the fat causes increased adipose LPL activity, directly lowering TG. Another possibility could be that less ANGPTL4/8 in the adipose tissue is available to block ANGPTL3/8-mediated inhibition of adipose LPL. This might shift FA uptake more toward skeletal muscle for oxidation, thereby decreasing TG. It is hard to know which, if either, of these mechanisms is correct, and further study will be required to address these possibilities.

In the case of the human ANGPTL8 knockout (121X) mutation, decreased circulating ANGPTL3/8 complex should result in decreased TG, which has been reported (82). The ANGPTL8 121X mutation should provide cardiovascular protection, as ANGPTL8 knockout mice have decreased TG and decreased fat mass (41). The mutation in humans, however, is so rare that even the extensive genetic study probing this question was underpowered (82). In retrospect, this is not surprising, as the ANGPTL8 121X mutation would have been extremely disadvantageous in a world of caloric insufficiency.

**Fig. 9.**
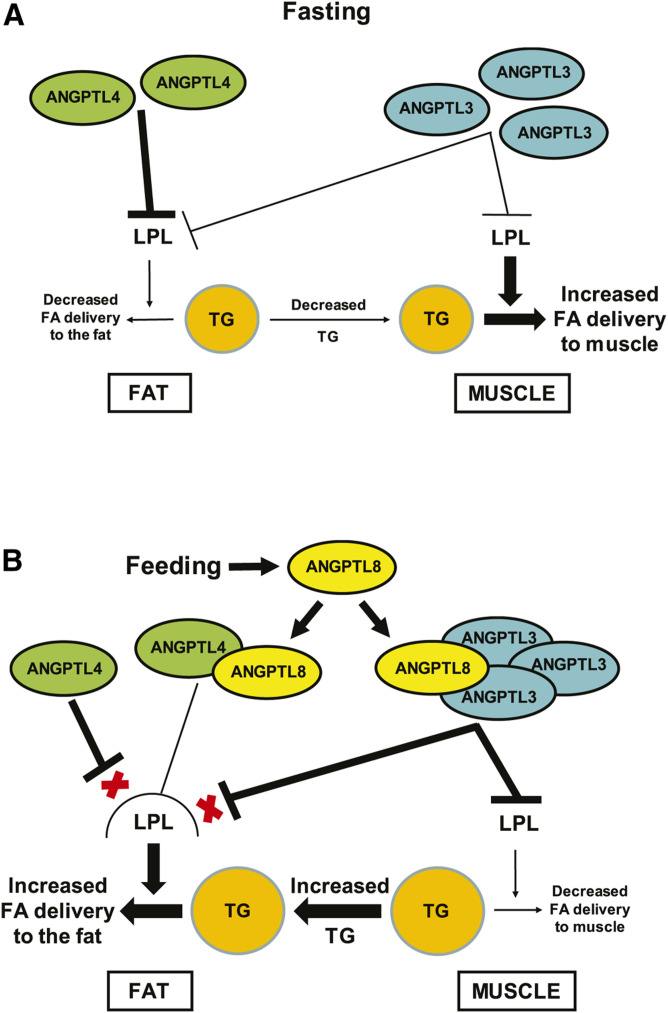
A possible model for how ANGPTL8 shifts FA toward adipose tissue after feeding. A: While fasting, ANGPTL8 levels are low. Localized ANGPTL4 inhibits adipose tissue LPL to minimize FA uptake into the fat for storage, and FAs are mainly taken up into skeletal muscle for use as energy. B: During feeding, ANGPTL8 forms a circulating complex with ANGPTL3 that increases its ability to inhibit LPL, thus minimizing FA uptake into skeletal muscle. ANGPTL8 also forms a mostly localized complex with ANGPTL4 in adipose tissue that decreases the ability of ANGPTL4 to inhibit LPL. The ANGPTL4/8 complex also protects LPL in the fat from inhibition by circulating ANGPTL3/8 and localized ANGPTL4 (denoted by red Xs), thereby preserving adipose tissue LPL activity to promote FA uptake into the fat for storage as TG.

Taken together, our data explain how ANGPTL8 responds to caloric intake to steer FA away from skeletal muscle and toward adipose tissue for storage as TG, as shown by the possible model in **Fig. 9**. Under fasting conditions (Fig. 9A), ANGPTL8 levels are low, and low levels of ANGPTL3/8 and ANGPTL4/8 complexes are made. As a result, LPL is inhibited locally in the fat by ANGPTL4, leading to minimal adipose tissue FA uptake, with most FA uptake occurring in skeletal muscle. Feeding dramatically changes this dynamic by stimulating release of ANGPTL8 in two different complexes to shift FA away from skeletal muscle and toward adipose tissue (Fig. 9B). Postprandial increases in insulin stimulate hepatic secretion of ANGPTL3/8, which potently inhibits LPL activity. The circulating ANGPTL3/8 complex reaches the skeletal muscle to inhibit LPL and prevent FA uptake. At the same time, postprandial increases in both insulin and GIP stimulate ANGPTL4/8 secretion from adipocytes. When ANGPTL8 is present in this localized complex with ANGPTL4, it drastically decreases the potency of ANGPTL4’s LPL-inhibitory activity. The increased localized ANGPTL4/8 in the adipose tissue not only preserves LPL activity but also blocks the ability of circulating ANGPTL3/8 and localized ANGPTL4 to inhibit LPL, with the net result of these actions being increased FA uptake into the adipose tissue for storage as TG.

When viewed holistically, it becomes apparent that the major metabolic problem in our developed world is that, unlike our ancestors, we hardly ever go through any periods of prolonged fasting. Instead, our constant feeding chronically increases our ANGPTL3/8 and ANGPTL4/8 levels. Increased levels of these complexes lead to elevated circulating TG and excessive FA storage in our adipose tissue, which in turn lead to obesity, hypertension, insulin resistance, and ultimately type 2 diabetes. Ironically, in a world of caloric abundance, the same ANGPTL8 protein that likely protected our ancestors from starvation now predisposes us to metabolic syndrome.

### Data availability

All study data are contained within the article and the supplementary data file. All primary mass spectrometry data have been deposited at PeptideAtlas (server name: ftp.peptideatlas.org) as follows: full URL, ftp://PASS01578:NM8576fr@ftp.peptideatlas.org/; data identifier, PASS01578; dataset type, SRM; dataset tag, ANGPTL; dataset title, SRM quantification of ANGPTL3/4/8 proteins.

## Supplementary Material

Supplemental Data
