# Supplementary material for: Angiopoietin-like protein 8 differentially regulates ANGPTL3 and ANGPTL4 during postprandial partitioning of fatty acids
Source: J Lipid Res. 2020 Jun 2;61(8):1203–20. doi: 10.1194/jlr.RA120000781 (PMC7397750; doi:10.1194/jlr.RA120000781)
Supplement: Supplemental Data [file supp_61_8_1203__index.html]

Angiopoietin-like protein 8 differentially regulates ANGPTL3 and ANGPTL4 during postprandial partitioning of fatty acids — ANGPTL8 differentially regulates ANGPTL3 and ANGPTL4 — Angiopoietin-like protein 8 differentially regulates ANGPTL3 and ANGPTL4 during postprandial partitioning of fatty acids — Supplemental Data 

# Angiopoietin-like protein 8 differentially regulates ANGPTL3 and ANGPTL4 during postprandial partitioning of fatty acids

## Supplemental Data

- Supplemental Information - Supplemental Tables 1-4 and supplemental Figures 1-7
