# Supplementary material for: Angiopoietin-like protein 8 differentially regulates ANGPTL3 and ANGPTL4 during postprandial partitioning of fatty acids
Source: J Lipid Res. 2020 Jun 2;61(8):1203–20. doi: 10.1194/jlr.RA120000781 (PMC7397750; doi:10.1194/jlr.RA120000781)
Supplement: Supplemental Data [file supp_RA120000781_159705_1_supp_527606_qs2qdy.pdf]

SUPPLEMENTAL INFORMATION

Angiopoietin-like protein 8 differentially regulates ANGPTL3 and ANGPTL4 during postprandial partitioning of fatty acids

Yan Q. Chen[1]*, Thomas G. Pottanat[1]*, Robert W. Siegel[1], Mariam Ehsani[1], Yue-Wei Qian[1], Eugene Y. Zhen[1], Ajit Regmi[1], William C. Roell[1], Haihong Guo[1], M. Jane Luo[1], Ruth E. Gimeno[1], Ferdinand van't Hooft[2], and Robert J. Konrad[1]**

[1]Lilly Research Laboratories, Eli Lilly and Company, Indianapolis, IN, USA
[2]Division of Cardiovascular Medicine, Department of Medicine Solna, Karolinska Institutet Karolinska University Hospital Solna, Stockholm, Sweden.

protein_name	Peptide location	sequence	Q1	Q3	dwelltime	Tr_original	transition_group	CE	relative_	decoy	isotype	prec_z	frg_type	frg_nr	frg_z	Frg_loss	RF Lens (V)	organism
ANGPTL3	239-252	HDGIPAEC[57]TTIYNR	549.7	423.2	11.3	3.3	1	19	0	0	Light	3	b	4	1		65	Human
ANGPTL3	239-252	HDGIPAEC[57]TTIYNR	549.7	452.2	11.3	3.3	1	10.25	0	0	Light	3	y	3	1	-36	65	Human
ANGPTL3	239-252	HDGIPAEC[57]TTIYNR	549.7	513.3	11.3	3.3	1	10.25	0	0	Light	3	MH	14	3		65	Human
ANGPTL3	239-252	HDGIPAEC[57]TTIYNR(13C6, 15N4)	553.1	423.2	11.3	3.3	2	19	0	0	Heavy	3	b	4	1		65	Human
ANGPTL3	239-252	HDGIPAEC[57]TTIYNR(13C6, 15N4)	553.1	462.2	11.3	3.3	2	10.25	0	0	Heavy	3	y	3	1		65	Human
ANGPTL3	239-252	HDGIPAEC[57]TTIYNR(13C6, 15N4)	553.1	516.7	11.3	3.3	2	10.25	0	0	Heavy	3	MH	14	3	-36	65	Human
ANGPTL3	378-387	DLVFSTWDHK	416.63	460.79	13.3	4.025	3	10.25	0	0	Light	3	y	7	2		55	Human
ANGPTL3	378-387	DLVFSTWDHK	416.63	510.32	13.3	4.025	3	10.25	0	0	Light	3	y	8	2		55	Human
ANGPTL3	378-387	DLVFSTWDHK(13C6, 15N2)	419.3	464.79	13.3	4.025	4	10.25	0	0	Heavy	3	y	7	2		55	Human
ANGPTL3	378-387	DLVFSTWDHK(13C6, 15N2)	419.3	514.32	13.3	4.025	4	10.25	0	0	Heavy	3	y	8	2		55	Human
ANGPTL3	132-137	ILLQQK	371.9	199.2	11.3	3.25	13	14	0	0	Light	2	a	2	1		59	Human
ANGPTL3	132-137	ILLQQK	371.9	227.2	11.3	3.25	13	12	0	0	Light	2	b	2	1		59	Human
ANGPTL3	132-137	ILLQQK	371.9	516.4	11.3	3.25	13	13	0	0	Light	2	y	4	1		59	Human
ANGPTL3	132-137	ILLQQK	371.9	629.4	11.3	3.25	13	13	0	0	Light	2	y	5	1		59	Human
ANGPTL3	132-137	ILLQQK(13C6, 15N2)	375.9	199.2	11.3	3.25	14	14	0	0	Heavy	2	a	2	1		59	Human
ANGPTL3	132-137	ILLQQK(13C6, 15N2)	375.9	227.2	11.3	3.25	14	12	0	0	Heavy	2	b	2	1		59	Human
ANGPTL3	132-137	ILLQQK(13C6, 15N2)	375.9	524.4	11.3	3.25	14	13	0	0	Heavy	2	y	4	1		59	Human
ANGPTL3	132-137	ILLQQK(13C6, 15N2)	375.9	637.4	11.3	3.25	14	13	0	0	Heavy	2	y	5	1		59	Human
ANGPTL3	177-187	DLLQTVEDQYK	676.5	201.2	23	4.2	15	34	0	0	Light	2	a	2	1		86	Human
ANGPTL3	177-187	DLLQTVEDQYK	676.5	229.1	23	4.2	15	24	0	0	Light	2	b	2	1		86	Human
ANGPTL3	177-187	DLLQTVEDQYK	676.5	682.3	23	4.2	15	18	0	0	Light	2	y	5	1		86	Human
ANGPTL3	177-187	DLLQTVEDQYK	676.5	882.5	23	4.2	15	20.5	0	0	Light	2	y	7	1		86	Human
ANGPTL3	177-187	DLLQTVEDQYK(13C6, 15N2)	680.5	201.2	23	4.2	16	34	0	0	Heavy	2	a	2	1		86	Human
ANGPTL3	177-187	DLLQTVEDQYK(13C6, 15N2)	680.5	229.1	23	4.2	16	24	0	0	Heavy	2	b	2	1		86	Human
ANGPTL3	177-187	DLLQTVEDQYK(13C6, 15N2)	680.5	690.3	23	4.2	16	18	0	0	Heavy	2	y	5	1		86	Human
ANGPTL3	177-187	DLLQTVEDQYK(13C6, 15N2)	680.5	890.5	23	4.2	16	20.5	0	0	Heavy	2	y	7	1		86	Human
ANGPTL4	64-71	SQLSALER	452.33	575.3	11.3	3.3	5	13.6	0	0	Light	2	y	5	1		58	Human
ANGPTL4	64-71	SQLSALER	452.33	688.4	11.3	3.3	5	12.8	0	0	Light	2	y	6	1		58	Human
ANGPTL4	64-71	SQLSALER(13C6, 15N4)	457.33	585.3	11.3	3.3	6	13.6	0	0	Heavy	2	y	5	1		58	Human
ANGPTL4	64-71	SQLSALER(13C6, 15N4)	457.33	698.4	11.3	3.3	6	12.8	0	0	Heavy	2	y	6	1		58	Human
ANGPTL4	97-110	VDPEVLHSLQTQLK	536.41	464.94	13.3	3.9	7	10.3	0	0	Light	3	y	12	3		67	Human
ANGPTL4	97-110	VDPEVLHSLQTQLK	536.41	697	13.3	3.9	7	10.3	0	0	Light	3	y	11	2		67	Human
ANGPTL4	97-110	VDPEVLHSLQTQLK(13C6, 15N2)	539.08	467.61	13.3	3.9	8	10.3	0	0	Heavy	3	y	12	3		67	Human
ANGPTL4	97-110	VDPEVLHSLQTQLK(13C6, 15N2)	539.08	701	13.3	3.9	8	10.3	0	0	Heavy	3	y	11	2		67	Human
ANGPTL8	133-138	LEVQLR	379.33	243.1	11.3	3.5	9	10.25	0	0	Light	2	b	2	1		56	Human
ANGPTL8	133-138	LEVQLR	379.33	515.27	11.3	3.5	9	10.25	0	0	Light	2	y	4	1		56	Human
ANGPTL8	133-138	LEVQLR	379.33	644.2	11.3	3.5	9	13.2	0	0	Light	2	y	5	1		56	Human
ANGPTL8	133-138	LEVQLR(13C6, 15N4)	384.33	243.05	11.3	3.5	10	10.25	0	0	Heavy	2	b	2	1		56	Human
ANGPTL8	133-138	LEVQLR(13C6, 15N4)	384.33	525.27	11.3	3.5	10	10.25	0	0	Heavy	2	y	4	1		56	Human
ANGPTL8	133-138	LEVQLR(13C6, 15N4)	384.33	654.2	11.3	3.5	10	13.2	0	0	Heavy	2	y	5	1		56	Human
ANGPTL8	148-153	EFEVLK	382.8	260.15	11.3	3.65	11	10.25	0	0	Light	2	y	2	1		55	Human
ANGPTL8	148-153	EFEVLK	382.8	388.1	11.3	3.65	11	10.25	0	0	Light	2	b	3	1	-18	55	Human
ANGPTL8	148-153	EFEVLK	382.8	487.2	11.3	3.65	11	10.25	0	0	Light	2	b	4	1	-18	55	Human
ANGPTL8	148-153	EFEVLK(13C6, 15N2)	386.8	268.15	11.3	3.65	12	10.25	0	0	Heavy	2	y	2	1		55	Human
ANGPTL8	148-153	EFEVLK(13C6, 15N2)	386.8	388.1	11.3	3.65	12	10.25	0	0	Heavy	2	b	3	1	-18	55	Human
ANGPTL8	148-153	EFEVLK(13C6, 15N2)	386.8	487.2	11.3	3.65	12	10.25	0	0	Heavy	2	b	4	1	-18	55	Human

Supplemental Table S1: ANGPTL protein MRM peptide characteristics

Integrated AUC, peptide ion ratios and calculated protein concentrations for experiments presented in Figure 1B.

	ANGPTL3_239-252			ANGPTL3_378-387			ANGPTL3		ANGPTL8_133-138			ANGPTL8_148-153			ANGPTL8	
	Unlabeled	SIL	Area ratio	Unlabeled	SIL	Area ratio	Averaged ratio	ANGTL3 (ng/ml)	Unlabeled	SIL	Area ratio	Unlabeled	SIL	Area ratio	Averaged ratio	ANGPTL8 (ng/ml)
Anti-ANGPTL3_IP_01	8.28E+05	4.05E+04	20.42	1.85E+06	8.26E+04	22.45	21.43	**222.5**	2.64E+05	5.07E+05	0.52	9.18E+04	1.75E+05	0.52	0.52	**2.08**
Anti-ANGPTL3_IP_02	8.38E+05	4.54E+04	18.46	1.94E+06	8.36E+04	23.17	20.82	**216.1**	2.52E+05	5.58E+05	0.45	9.07E+04	1.70E+05	0.53	0.49	**1.96**
Anti-ANGPTL3_IP_03	8.12E+05	4.42E+04	18.36	1.98E+06	8.65E+04	22.92	20.64	**214.3**	2.52E+05	5.44E+05	0.46	9.22E+04	1.82E+05	0.51	0.49	**1.93**
SD			1.16			0.37	0.42	**4.3**			0.04			0.01	0.02	**0.08**
Anti-ANGPTL8_IP_01	6.59E+04	6.48E+04	1.02	1.14E+05	9.57E+04	1.19	1.10	**11.4**	5.34E+05	5.86E+05	0.91	2.03E+05	2.23E+05	0.91	0.91	**3.63**
Anti-ANGPTL8_IP_02	5.47E+04	7.14E+04	0.77	1.08E+05	1.11E+05	0.97	0.87	**9.0**	4.86E+05	6.69E+05	0.73	1.79E+05	2.55E+05	0.70	0.71	**2.84**
Anti-ANGPTL8_IP_03	7.42E+04	7.31E+04	1.02	1.31E+05	1.04E+05	1.26	1.14	**11.8**	6.16E+05	6.36E+05	0.97	2.24E+05	2.42E+05	0.93	0.95	**3.77**
SD			0.14			0.15	0.14	**1.5**			0.13			0.13	0.13	**0.50**
Anti-ANGPTL4_IP_01									4.65E+04	5.26E+05	0.09	1.94E+04	2.19E+05	0.09	0.09	**0.35**
Anti-ANGPTL4_IP_02									4.25E+04	5.41E+05	0.08	1.91E+04	2.21E+05	0.09	0.08	**0.33**
Anti-ANGPTL4_IP_03									5.04E+04	5.54E+05	0.09	1.77E+04	2.31E+05	0.08	0.08	**0.33**
SD											0.01			0.01	0.00	**0.01**

	ANGPTL4_64-71			ANGPTL4_97-110			ANGPTL4	
	Unlabeled	SIL	Area ratio	Unlabeled	SIL	Area ratio	Averaged ratio	ANGPTL4 (ng/ml)
Anti-ANGPTL3_IP_01								
Anti-ANGPTL3_IP_02								
Anti-ANGPTL3_IP_03								
SD								
Anti-ANGPTL8_IP_01	1.88E+04	3.73E+05	0.05	6.45E+04	7.42E+05	0.09	0.07	**0.59**
Anti-ANGPTL8_IP_02	1.75E+04	4.11E+05	0.04	6.17E+04	8.63E+05	0.07	0.06	**0.49**
Anti-ANGPTL8_IP_03	2.09E+04	4.12E+05	0.05	6.77E+04	7.96E+05	0.08	0.07	**0.58**
SD			0.005			0.01	0.01	**0.06**
Anti-ANGPTL4_IP_01	5.85E+04	3.56E+05	0.16	1.98E+05	8.68E+05	0.23	0.20	**1.69**
Anti-ANGPTL4_IP_02	5.83E+04	3.55E+05	0.16	1.95E+05	8.36E+05	0.23	0.20	**1.71**
Anti-ANGPTL4_IP_03	5.72E+04	3.46E+05	0.17	1.95E+05	8.39E+05	0.23	0.20	**1.71**
SD			0.000			0.003	0.001	**0.01**

Supplemental Table S2: MRM peptide AUCs for ANGPTL protein immunoprecipitations. Integrated AUC values for each peptide ion and calculated ANGPTL protein concentration are listed.

Integrated AUC, peptide ion ratios and proteins ratios for recombinant protein complexes presented in Table 1.

	ANGPTL3_239-252			ANGPTL3_378-387			ANGPTL3	ANGPTL8_133-138			ANGPTL8_148-153			ANGPTL8	ANGPTL4_64-71			ANGPTL4_97-110			ANGPTL4	Stoichiometry	
	Unlabeled	SIL	Area ratio	Unlabeled	SIL	Area ratio	Averaged ratio	Unlabeled	SIL	Area ratio	Unlabeled	SIL	Area ratio	Averaged ratio	Unlabeled	SIL	Area ratio	Unlabeled	SIL	Area ratio	Averaged ratio	ANGPTL3/8	ANGPTL4/8
recombinant Angptl3/8_01	8.37E+06	8.72E+05	9.60	1.75E+07	1.35E+06	12.96	11.28	2.13E+07	5.84E+06	3.64	1.09E+07	3.75E+06	2.92	3.28								**3.4**	
recombinant Angptl3/8_02	9.14E+06	9.94E+05	9.19	1.82E+07	1.32E+06	13.74	11.47	2.16E+07	5.93E+06	3.64	1.12E+07	3.70E+06	3.04	3.34								**3.4**	
recombinant Angptl3/8_03	6.57E+06	8.15E+05	8.06	1.87E+07	1.39E+06	13.45	10.75	1.92E+07	5.96E+06	3.23	1.07E+07	3.68E+06	2.92	3.08								**3.5**	
SD			0.80			0.40	0.37			0.24			0.07	0.14								0.03	
recombinant Angptl4/8_01								5.59E+06	6.31E+06	0.88	2.83E+06	3.85E+06	0.74	0.81	3.70E+06	5.20E+06	0.71	8.67E+06	1.07E+07	0.81	0.76		**0.9**
recombinant Angptl4/8_02								4.49E+06	6.61E+06	0.68	2.21E+06	3.90E+06	0.57	0.62	2.88E+06	5.36E+06	0.54	7.12E+06	1.07E+07	0.67	0.60		**1.0**
recombinant Angptl4/8_03								5.24E+06	7.07E+06	0.74	2.77E+06	4.44E+06	0.62	0.68	3.89E+06	5.71E+06	0.68	8.75E+06	9.54E+06	0.92	0.80		**1.2**
SD										0.11			0.09	0.10			0.09			0.13	0.11		0.13

Supplemental Table S3: MRM peptide AUCs for recombinant ANGPTL3/8 and ANGPTL4/8 protein complexes. Integrated AUC values for each peptide ion and the protein ratios for the recombinant ANGPTL3/8 and ANGPTL4/8 complexes are listed.

Integrated AUC, peptide ion ratios and proteins ratios for endogenous protein complexes presented in Table 1.

	ANGPTL3_132-137			ANGPTL3_177-187			ANGPTL3	ANGPTL8_133-138			ANGPTL8_148-153			ANGPTL8	ANGPTL4_64-71			ANGPTL4_97-110			ANGPTL4	Stoichiometry	
	Unlabeled	SIL	Area ratio	Unlabeled	SIL	Area ratio	Averaged ratio	Unlabeled	SIL	Area ratio	Unlabeled	SIL	Area ratio	Averaged ratio	Unlabeled	SIL	Area ratio	Unlabeled	SIL	Area ratio	Averaged ratio	ANGPTL3/8	ANGPTL4/8
Endogenous Angptl3/8_01	5.82E+05	4.57E+05	1.27	3.46E+04	2.58E+04	1.34	1.31	1.98E+05	5.40E+05	0.37	9.78E+04	2.60E+05	0.38	0.37								**3.5**	
Endogenous Angptl3/8_02	6.03E+05	4.80E+05	1.25	3.37E+04	2.66E+04	1.27	1.26	2.18E+05	5.87E+05	0.37	9.39E+04	2.64E+05	0.36	0.36								**3.4**	
SD			0.02			0.05	0.03			0.004			0.01	0.01								0.08	
Endogenous Angptl4/8_01								3.01E+04	4.83E+05	0.062	8.47E+03	1.47E+05	0.058	0.060	7.10E+03	1.75E+05	0.041	3.52E+04	6.41E+05	0.055	0.048		**0.8**
Endogenous Angptl4/8_02								2.64E+04	4.45E+05	0.059	9.18E+03	1.48E+05	0.062	0.061	1.20E+04	2.80E+05	0.043	2.99E+04	5.70E+05	0.052	0.048		**0.8**
SD										0.002			0.003	0.00			0.002			0.002	0.00		0.01

Supplemental Table S4: MRM peptide AUCs for endogenous ANGPTL3/8 and ANGPTL4/8 protein complexes. Integrated AUC values for each peptide ion and the protein ratios for the endogenous ANGPTL3/8 and ANGPTL4/8 complexes are listed.

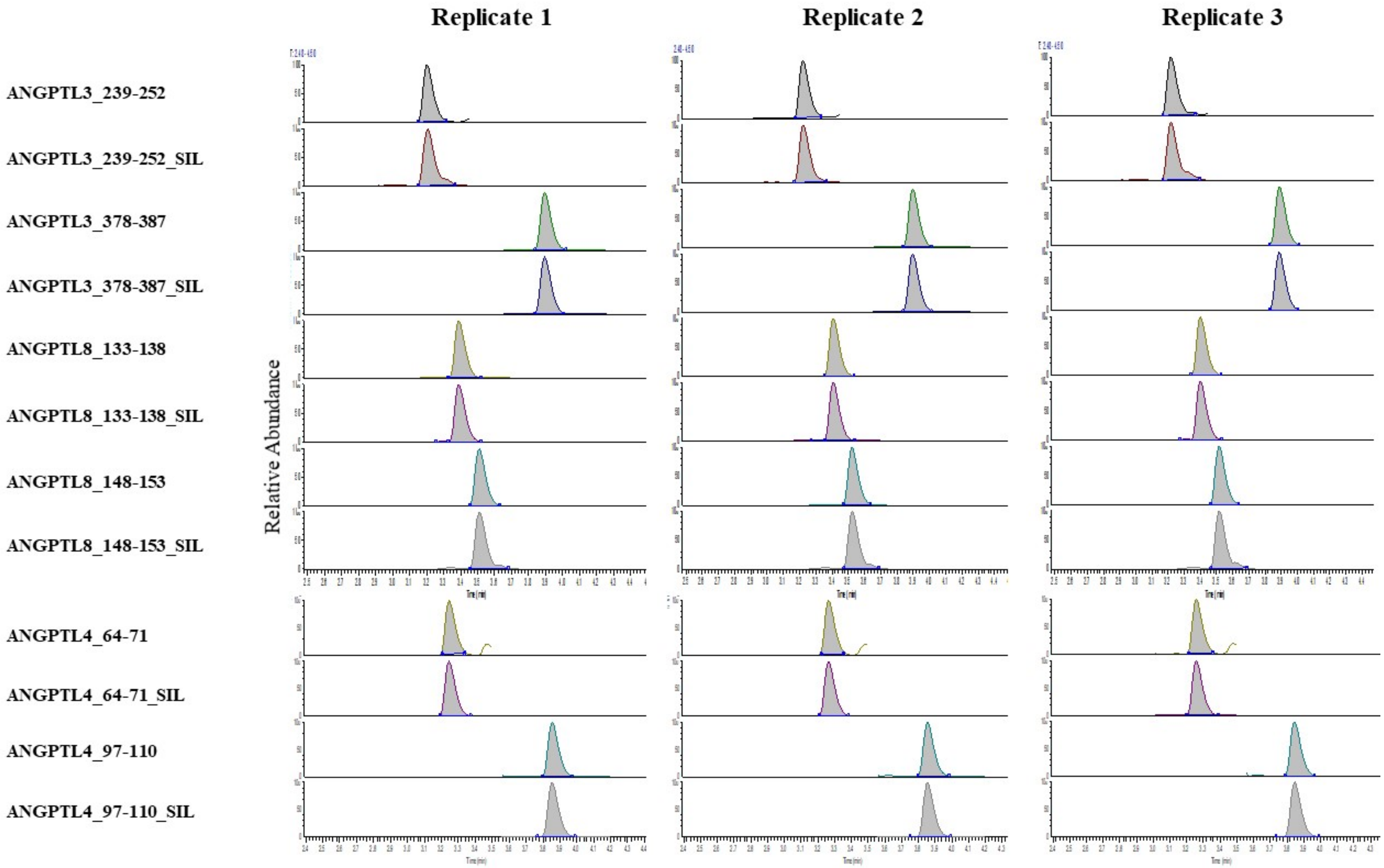

Supplemental Figure S1: Chromatograms of protein ions detected in ANGPTL8 immunoprecipitation experiments. MRM data were quantified using Thermo Xcalibur (version 4.2.47) with ICIS peak detection algorithm. The following parameters were used for peak integration: Smoothing points: 7; Baseline window: 20; Area noise factor: 5; Peak noise factor: 10. Two peptides per protein were monitored. SIL peptides (0.2 pmole) were spiked into each replicate after digestion. The ratio between endogenous peptide and the corresponding SIL peptide was calculated and the amount of protein detected was determined using the molecular weight. All analyses were performed in triplicate. The SIL peptides used for quantitation of ANGPTL8 were: 133-138 and 148-153, those used for ANGPTL4 were 64-71 and 97-110, and those used for ANGPTL3 were 239-252 and 378-387. The Y-axis shows relative abundance, and the X-axis shows retention time in minutes, with grey shading indicating the integrated area. The integrated AUC values for each peptide ion and calculated protein concentrations are listed in Supplemental Table 2.

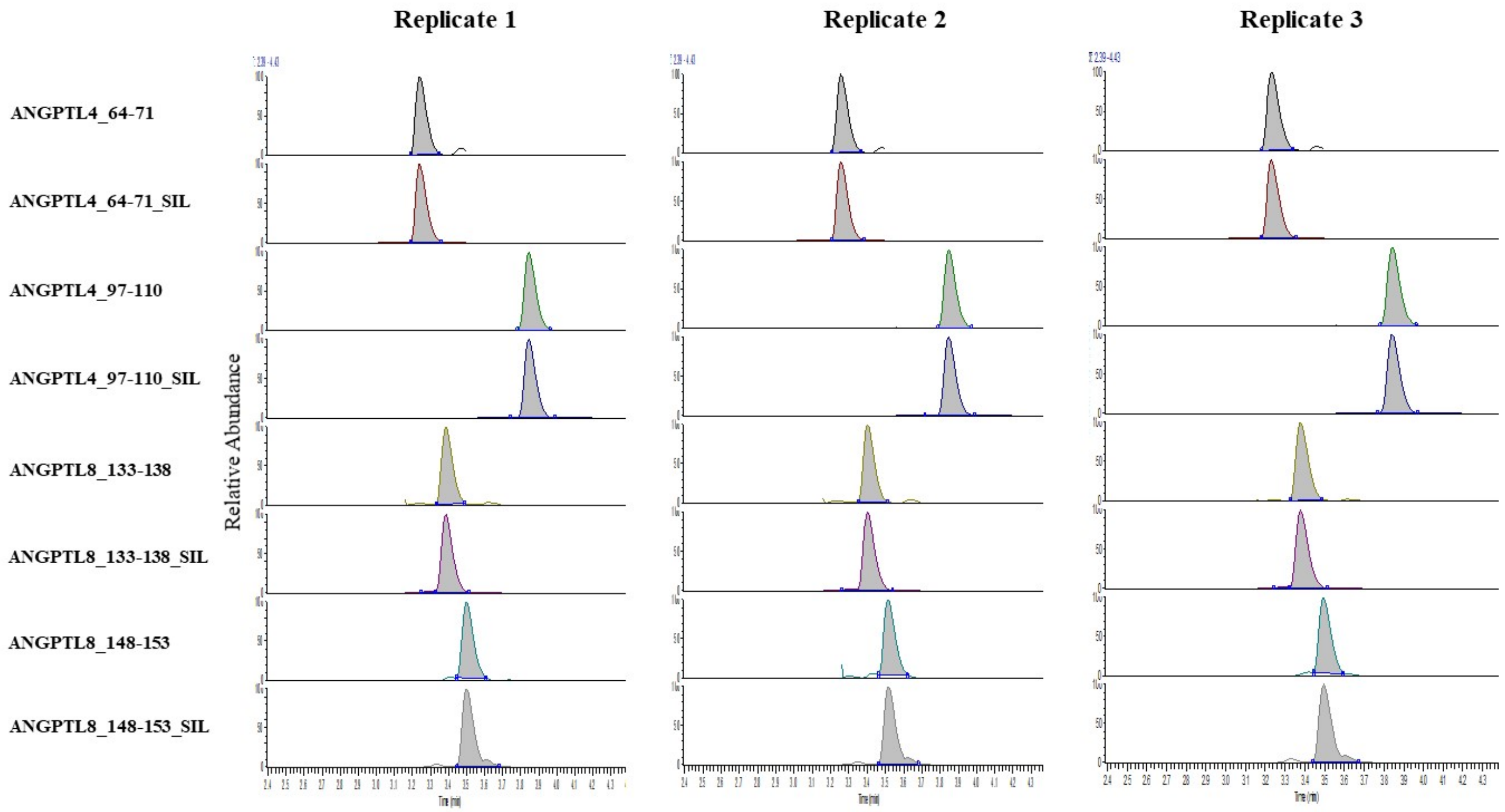

Supplemental Figure S2: Chromatograms of protein ions detected in ANGPTL4 immunoprecipitation experiments. MRM data were quantified using Thermo Xcalibur (version 4.2.47) with ICIS peak detection algorithm. The following parameters were used for peak integration: Smoothing points: 7; Baseline window: 20; Area noise factor: 5; Peak noise factor: 10. Two peptides per protein were monitored. SIL peptides (0.2 pmole) were spiked into each replicate after digestion. The ratio between endogenous peptide and the corresponding SIL peptide was calculated and the amount of protein detected was determined using the molecular weight. All analyses were performed in triplicate. The SIL peptides used for quantitation of ANGPTL4 were: 64-71 and 97-110, and those used for ANGPTL8 were 133-138 and 148-153. The Y-axis shows relative abundance, and the X-axis shows retention time in minutes, with grey shading indicating the integrated area. The integrated AUC values for each peptide ion and calculated protein concentrations are listed in Supplemental Table 2.

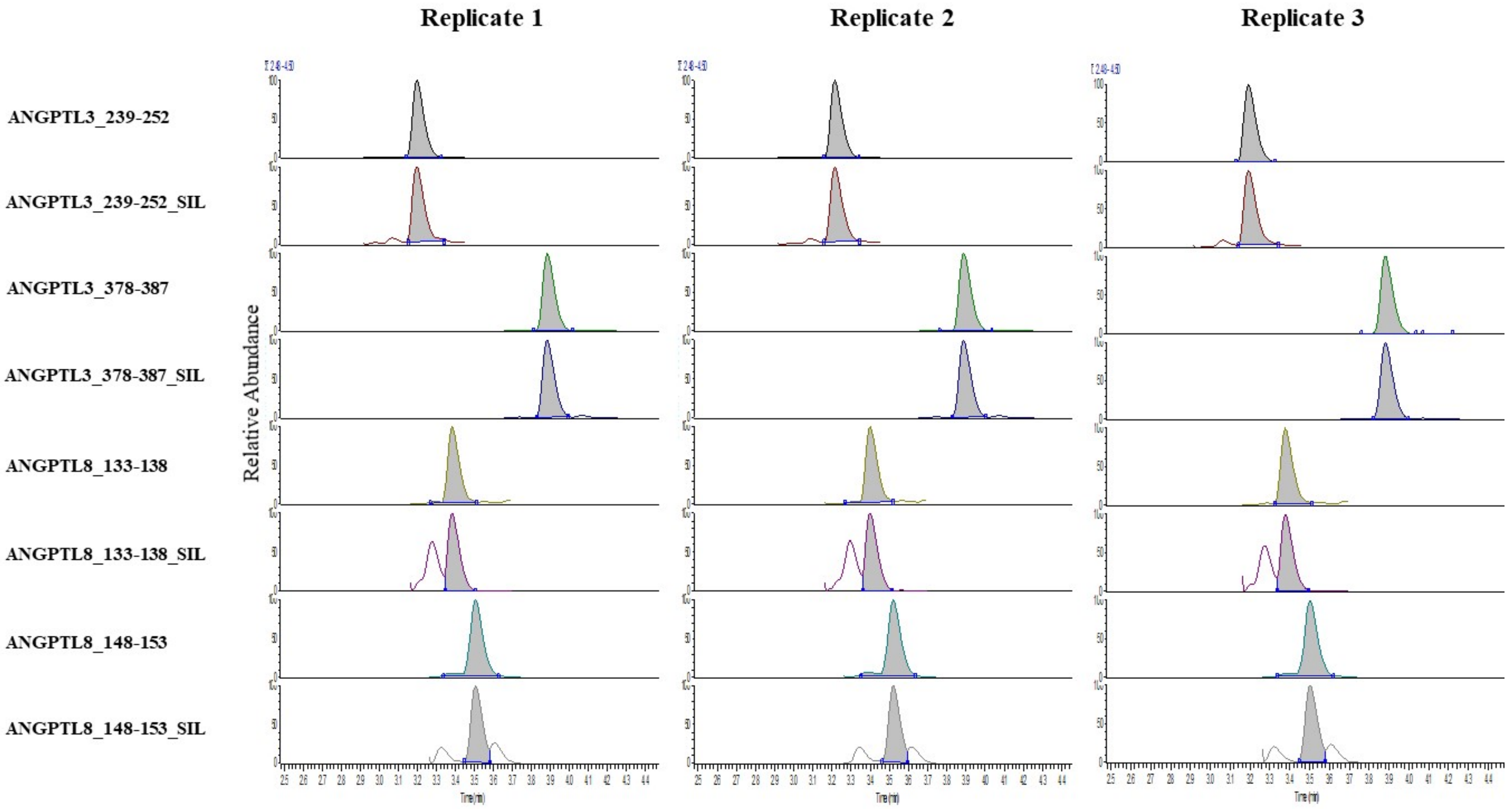

Supplemental Figure S3: Chromatograms of protein ions detected in ANGPTL3 immunoprecipitation experiments. MRM data were quantified using Thermo Xcalibur (version 4.2.47) with ICIS peak detection algorithm. The following parameters were used for peak integration: Smoothing points: 7; Baseline window: 20; Area noise factor: 5; Peak noise factor: 10. Two peptides per protein were monitored. SIL peptides (0.2 pmole) were spiked into each replicate after digestion. The ratio between endogenous peptide and the corresponding SIL peptide was calculated and the amount of protein detected was determined using the molecular weight. All analyses were performed in triplicate. The SIL peptides used for quantitation of ANGPTL3 were 239-252 and 378-387, and those used for ANGPTL8 were 133-138 and 148-153. The Y-axis shows relative abundance, and the X-axis shows retention time in minutes, with grey shading indicating the integrated area. The integrated AUC values for each peptide ion and calculated protein concentrations are listed in Supplemental Table 2.

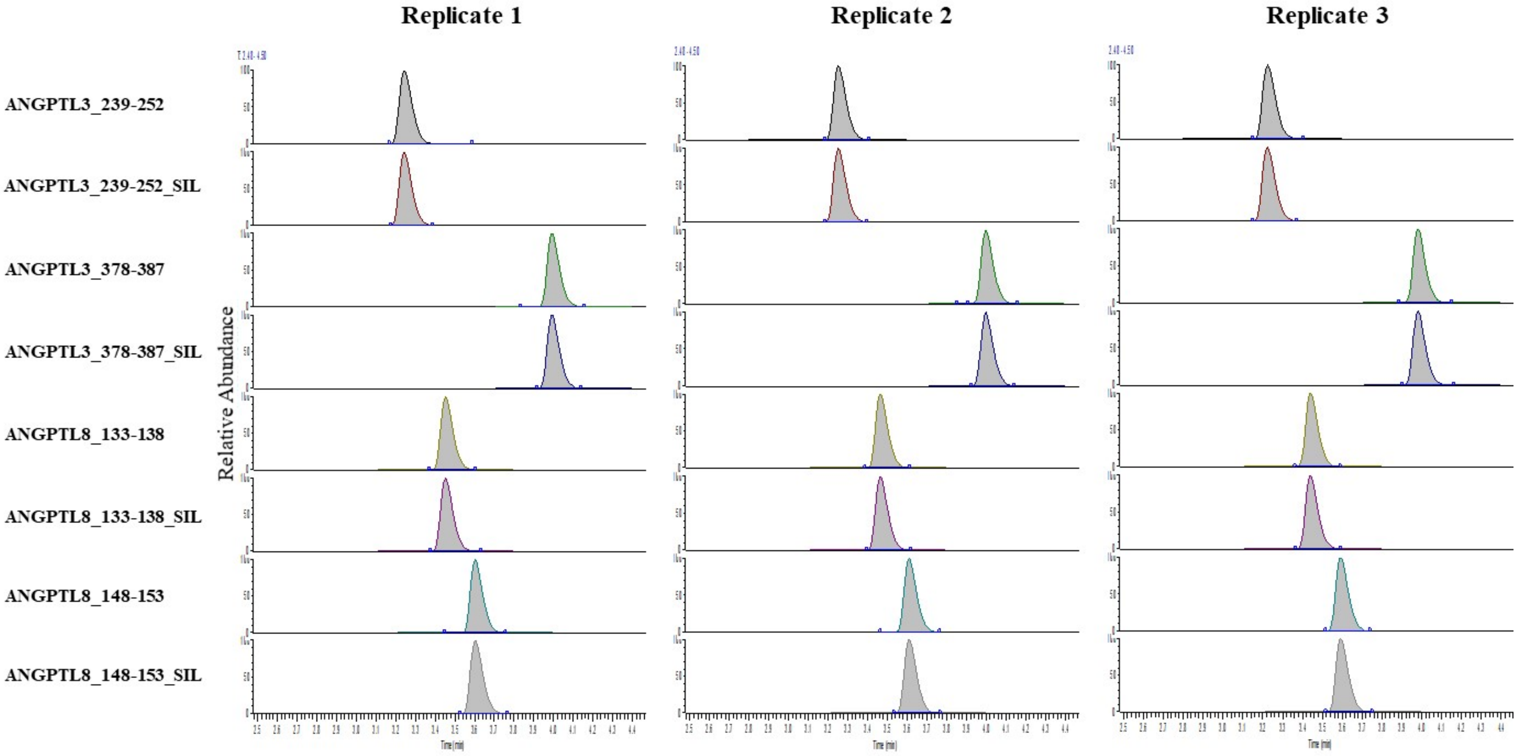

Supplemental Figure S4: Chromatograms for ANGPTL protein ions from recombinant ANGPTL3/8 digest. The peak detection methods are the same as described in Supplemental Figures 1-3. All analyses were performed in triplicate from a single protein production preparation. Two peptides per protein were monitored. The SIL peptides used for quantitation were 239-252 and 378-387 for ANGPTL3 and 133-138 and 148-153 for ANGPTL8. The Y-axis shows the relative abundance, and the X-axis shows retention time in minutes, with grey shading indicating the integrated area. The integrated AUC values for each peptide ion and the protein ratios for the complex are listed in Supplemental Table 2.

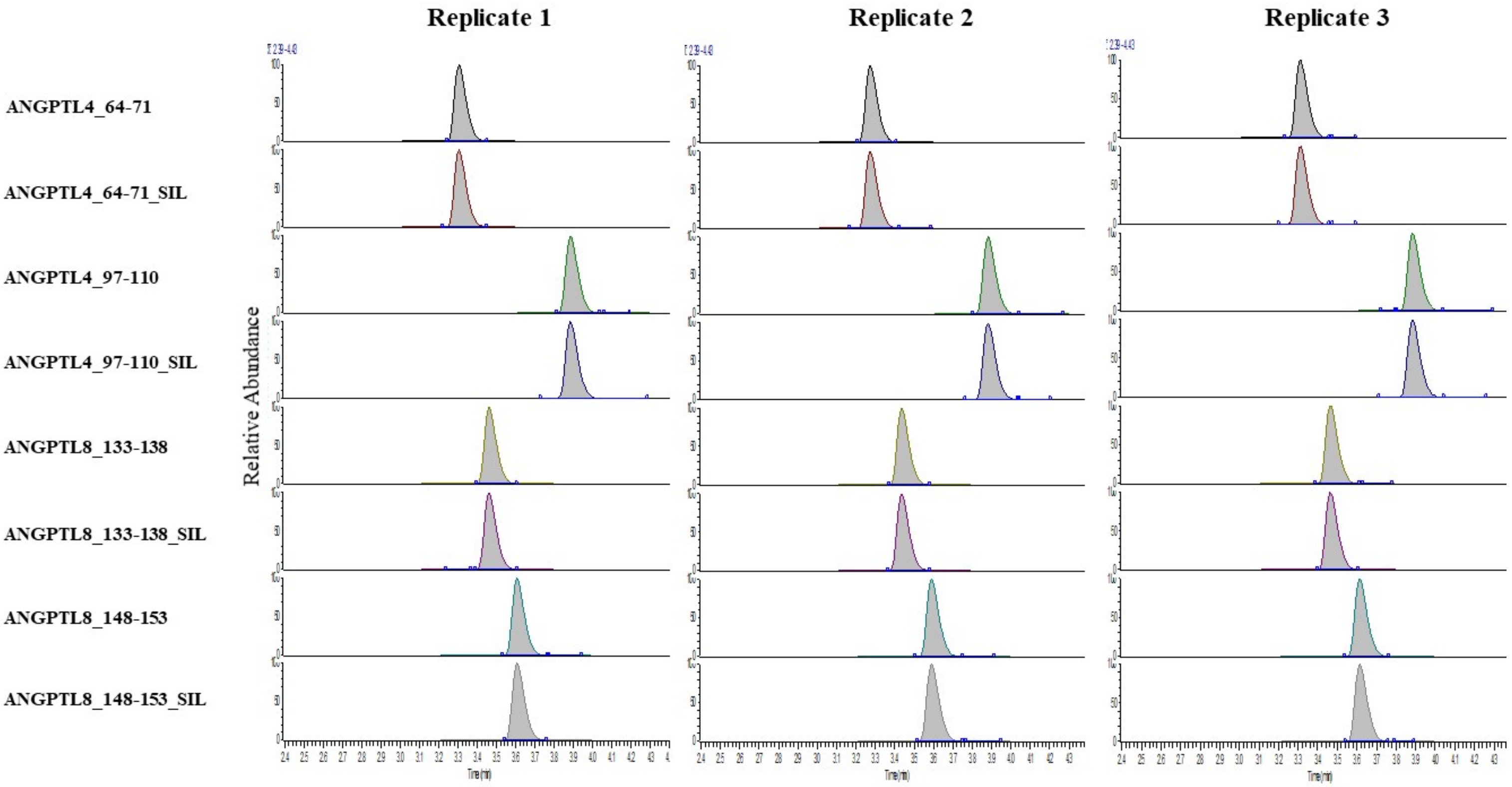

Supplemental Figure S5: Chromatograms for ANGPTL protein ions from recombinant ANGPTL4/8 digest. The peak detection methods are the same as described in Supplemental Figures 1-3. All analyses were performed in triplicate from a single protein production preparation. Two peptides per protein were monitored. The SIL peptides used for quantitation were 64-71 and 97-110 for ANGPTL4 and 133-138 and 148-153 for ANGPTL8. The Y-axis shows the relative abundance, and the X-axis shows retention time in minutes, with grey shading indicating the integrated area. The integrated AUC values for each peptide ion and the protein ratios for the complex are listed in Supplemental Table 2.

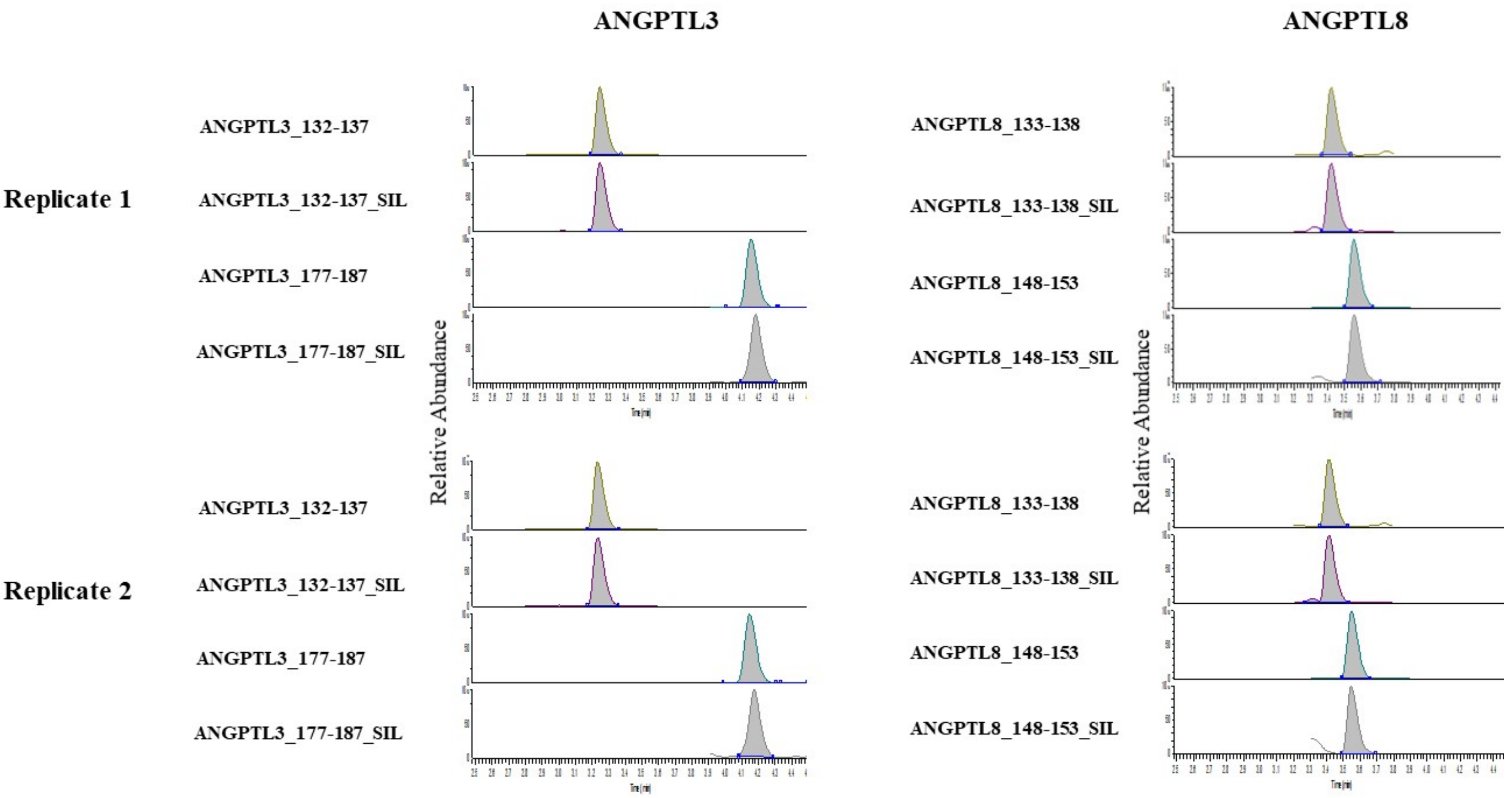

Supplemental Figure S6: Chromatograms for ANGPTL protein ions from endogenous ANGPTL3/8 digest. The peak detection methods are the same as described in Supplemental Figures 1-3. All analyses were performed in duplicate from a sample composed of a pool of sera from 20 healthy donors. Two peptides per protein were monitored. The SIL peptides used for quantitation were 132-137 and 177-187 for ANGPTL3 and 133-138 and 148-153 for ANGPTL8. The Y-axis shows the relative abundance, and the X-axis shows retention time in minutes, with grey shading indicating the integrated area. The integrated AUC values for each peptide ion and the protein ratios for the complex are listed in Supplemental Table 2.

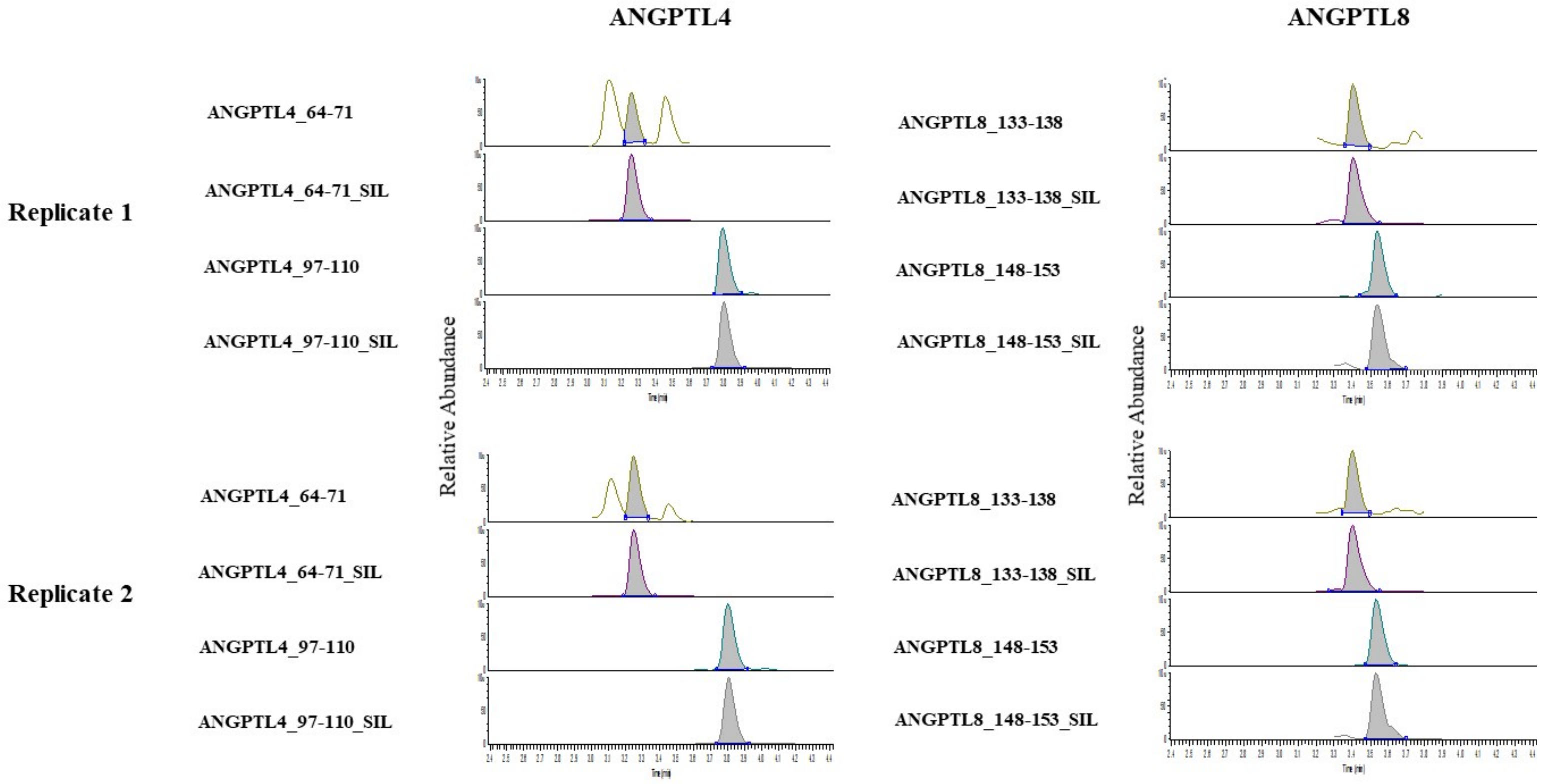

Supplemental Figure S7: Chromatograms for ANGPTL protein ions from endogenous ANGPTL4/8 digest. The peak detection methods are the same as described in Supplemental Figures 1-3. All analyses were performed in duplicate from a sample composed of a pool of sera from 20 healthy donors. Two peptides per protein were monitored. The SIL peptides used for quantitation were 64-71 and 97-110 for ANGPTL4 and 133-138 an 148-153 for ANGPTL8. The Y-axis shows the relative abundance, and the X-axis shows retention time in minutes, with grey shading indicating the integrated area. The integrated AUC values for each peptide ion and the protein ratios for the complex are listed in Supplemental Table 2.
